# Glycated ACE2 receptor in diabetes: open door for SARS-COV-2 entry in cardiomyocyte

**DOI:** 10.1186/s12933-021-01286-7

**Published:** 2021-05-07

**Authors:** Nunzia D’Onofrio, Lucia Scisciola, Celestino Sardu, Maria Consiglia Trotta, Marisa De Feo, Ciro Maiello, Pasquale Mascolo, Francesco De Micco, Fabrizio Turriziani, Emilia Municinò, Pasquale Monetti, Antonio Lombardi, Maria Gaetana Napolitano, Federica Zito Marino, Andrea Ronchi, Vincenzo Grimaldi, Anca Hermenean, Maria Rosaria Rizzo, Michelangela Barbieri, Renato Franco, Carlo Pietro Campobasso, Claudio Napoli, Maurizio Municinò, Giuseppe Paolisso, Maria Luisa Balestrieri, Raffaele Marfella

**Affiliations:** 1 Department of Precision Medicine, University of Campania L. Vanvitelli, Naples, Italy; 2 Department of Advanced Medical and Surgical Sciences, University of Campania L. Vanvitelli, Piazza Miraglia, 2, 80138 Naples, Italy; 3 Department of Experimental Medicine, University of Campania “Luigi Vanvitelli”, Naples, Italy; 4 Department of Cardio-Thoracic Sciences, University of Campania “Luigi Vanvitelli”, Naples, Italy; 5 Unit of Cardiac Surgery and Transplants, AORN Ospedali dei Colli-Monaldi Hospital, 80131 Naples, Italy; 6 Department of Experimental Medicine Forensic Pathology Service, University of Campania L. Vanvitelli, Naples, Italy; 7 Department of Forensic, Evaluative and Necroscopic Medicine, ASL Napoli 2 NORD, Naples, Italy; 8 Department of Mental and Physical Health and Preventive Medicine, University of Campania L. Vanvitelli, Naples, Italy; 9 Institute of Life Science, Vasile Goldis Western University, Arad, Romania; 10 Mediterranea Cardiocentro, Naples, Italy

**Keywords:** COVID-19, SARS-CoV-2, Cardiomyocyte, Diabetes, Heart, ACE2

## Abstract

**Rationale:**

About 50% of hospitalized coronavirus disease 2019 (COVID-19) patients with diabetes mellitus (DM) developed myocardial damage. The mechanisms of direct SARS-CoV-2 cardiomyocyte infection include viral invasion via ACE2-Spike glycoprotein-binding. In DM patients, the impact of glycation of ACE2 on cardiomyocyte invasion by SARS-CoV-2 can be of high importance.

**Objective:**

To evaluate the presence of SARS-CoV-2 in cardiomyocytes from heart autopsy of DM cases compared to Non-DM; to investigate the role of DM in SARS-COV-2 entry in cardiomyocytes.

**Methods and results:**

We evaluated consecutive autopsy cases, deceased for COVID-19, from Italy between Apr 30, 2020 and Jan 18, 2021. We evaluated SARS-CoV-2 in cardiomyocytes, expression of ACE2 (total and glycosylated form), and transmembrane protease serine protease-2 (TMPRSS2) protein. In order to study the role of diabetes on cardiomyocyte alterations, independently of COVID-19, we investigated ACE2, glycosylated ACE2, and TMPRSS2 proteins in cardiomyocytes from DM and Non-DM explanted-hearts. Finally, to investigate the effects of DM on ACE2 protein modification, an in vitro glycation study of recombinant human ACE2 (hACE2) was performed to evaluate the effects on binding to SARS-CoV-2 Spike protein. The authors included cardiac tissue from 97 autopsies. DM was diagnosed in 37 patients (38%). Fourth-seven out of 97 autopsies (48%) had SARS-CoV-2 RNA in cardiomyocytes. Thirty out of 37 DM autopsy cases (81%) and 17 out of 60 Non-DM autopsy cases (28%) had SARS-CoV-2 RNA in cardiomyocytes. Total ACE2, glycosylated ACE2, and TMPRSS2 protein expressions were higher in cardiomyocytes from autopsied and explanted hearts of DM than Non-DM. In vitro exposure of monomeric hACE2 to 120 mM glucose for 12 days led to non-enzymatic glycation of four lysine residues in the neck domain affecting the protein oligomerization.

**Conclusions:**

The upregulation of ACE2 expression (total and glycosylated forms) in DM cardiomyocytes, along with non-enzymatic glycation, could increase the susceptibility to COVID-19 infection in DM patients by favouring the cellular entry of SARS-CoV2.

**Supplementary Information:**

The online version contains supplementary material available at 10.1186/s12933-021-01286-7.

## The potential translational impact of the study results

In patients with diabetes mellitus, the upregulation of ACE2 expression in cardiomyocytes, together with non-enzymatic glycation favouring protein oligomerization, could increase the susceptibility to COVID-19 infection and worse prognosis. However, the control of the expression of ACE2 and its glycated form could represent a therapeutic target to prevent COVID-19 infection and worse prognosis in patients with diabetes.

## Introduction

The coronavirus disease-19 (COVID-19), caused by the RNA single-stranded enveloped virus of severe acute respiratory syndrome (SARS)-CoV-2, has a significant impact on the cardiovascular (CV) system by direct myocardial damage [1]. Indeed, a considerable number of hospitalized COVID-19 patients could develop cardiac injury (24.4%), with a consequent higher rate of mortality (72.6%), [2]. Notably, among hospitalized COVID-19 patients with diabetes (DM), about half of them developed myocardial damage [3]. Indeed, DM is very common among hospitalized COVID-19 patients, has a significant impact on the treatment [4], and negatively influences clinical outcomes in affected patients [5–7]. However, specific therapies to prevent coagulopathies, over-inflammation, and hyperglycemia may represent a valid therapeutic option for treating asymptomatic and non-critically ill COVID-19 patients with DM as critically-ill DM patients [8–12].

In this context, the impact of hyperglycemia in the progression and deterioration of heart function in COVID-19 patients is currently of great importance. Indeed, in DM patients, the severity of SARS-CoV-2 infection has been attributed to impaired innate and adaptive immunity, upregulation of ACE2, and potential changes in the glycation of ACE2 [4, 13]. Intriguingly, SARS-CoV-2 could invade the myocardium via ACE2-Spike binding and by the Spike priming by host cell transmembrane protease serine protease-2 (TMPRSS2), [14]. TMPRSS2 entails proteolytical protein cleavage and folding to a post-fusion conformation coupled with host cell–virus membrane fusion and cytosolic release of viral RNA [14, 15]. Thus, the susceptibility to SARS-CoV-2 infection primarily depends on the affinity of the Spike receptor‐binding domain (RBD) to ACE2 in target tissues [16]. The combined analysis of glycomics-informed glycoproteomics and bioinformatics of variants with molecular dynamics simulations highlighted roles for glycosylation in sterically masking polypeptide epitopes [17]. This could influence the conformation of ACE2, and modulating Spike-ACE2 interactions [17]. In this regard, the avidity effects during the interactions of the SARS-CoV-2 Spike with ACE2 are more potent for engineered trimeric and dimeric soluble ACE2 than the monomeric subunit [18].

Furthermore, the entry of the SARS-COV-2 into the host cells requires the cleavage of the S protein at the junction between the S1 and S2 subunits by TMPRSS2 to promote pathogenicity [19]. Notably, the recruitment of corin/TMPRSS10 by SARS-CoV-2 in cardiomyocytes could be the cause of the myocardial damage reported in COVID-19 patients [20]. However, SARS-COV-2 genome and proteins have been provided in cardiac samples of COVID-19 patients [21]. Conversely, infected cells and virions in cells can shed a substantial amount of free soluble S1 subunits with intact RBD domains [22]. Then, these S1 subunits can bind to ACE2, thus inducing ACE2 downregulation, which could cause a reduced viral infectivity towards the neighboring cells by decreasing the number of ACE2 receptor molecules on their surface [22].

Moreover, in this context, we might speculate that glycosylation might modulate the affinity of protein interactions and immune/inflammatory signaling pathways. Thus, it is relevant to elucidate the impact of ACE2 glycosylation concerning its binding to SARS-CoV-2 Spike. In this regard, the non-enzymatic glycation exacerbated by hyperglycemia is associated with long-term DM complications, including retinopathy, nephropathy, neuropathy, and cardiomyopathy [23].

Moreover, the non-enzymatic glycation of proteins interferes with their normal functions by altering molecular conformation and enzymatic activity and interfering with receptor functioning [23]. No evidence demonstrates hyperglycemia’s role on the expression of glycosylated ACE2 in cardiomyocytes and on the non-enzymatic glycation of ACE2, which could impact its binding to SARS-CoV-2 Spike. Furthermore, these adverse molecular effects induced by hyperglycemia might be behind DM’s effects on worsening the prognosis of COVID-19 patients and needs to be elucidated.

In the present study, to elucidate the pathogenesis of myocardial injury by COVID-19 in DM patients, we evaluated the presence of SARS-CoV-2 and expression levels of glycosylated ACE2 in cardiomyocytes from the autopsy of COVID-19 patients. Moreover, to investigate the ACE2 expression in DM people, independently from SARS-COV-2 infection, we evaluated the glycosylated ACE2 levels in DM and non-diabetes (Non-DM) explanted hearts from patients without COVID-19. Finally, we used high-resolution mass spectrometry and surface plasmon resonance (SPR) to evaluate the possible effect of in vitro high-glucose exposure on the structural conformation of recombinant human ACE2 (hACE2) monomer and binding to SARS-CoV-2 Spike immobilized dimer [24, 25].

## Material and methods

### Autopsy study cohort and tissue sampling analysis

Consecutively, deceased individuals with diagnosed SARS-CoV-2 infection were autopsied at the Institute of Legal Medicine at “Azienda Sanitaria Locale Napoli” in Italy between Apr 30 and Dec 18, 2020. The diagnosis was confirmed post mortem by a quantitative reverse transcriptase-polymerase chain reaction to detect SARS-CoV-2 RNA and performed from pharyngeal swabs. The median (interquartile range) age of the 97 individuals was 63 (69–75) years, and 60 (61.8%) were male. The local ethics committee approved this study of the Vanvitelli University. The investigation complied with the principles outlined in the Declaration of Helsinki. We included the cases in the autopsy study of the first consecutive 97 individuals who had died of SARS-CoV-2 infection in Campania, Italy, which reported cause of death. We divided the cases into two main groups according to whether patients did or did not have pre-deceased DM according to American Diabetes Association (ADA) guidelines. Authors collected cardiac tissue during autopsy with median post-mortem intervals of 3.0 (interquartile range, 2.0–4.3) days. The authors took two tissue specimens from the left ventricle and either snap-frozen in liquid nitrogen or fixed them in formalin for subsequent analysis.

#### SARS-CoV-2 RNA in situ hybridization

We performed the SARS-CoV-2 RNA in situ hybridization (ISH) on formalin-fixed paraffin-embedded (FFPE) samples. The authors detected SARS-CoV-2 RNA using Ready-to-use reagents from RNAscope 2.5 LS Reagent Kit-BROWN and the V-nCoV2019-S probe (advanced cell diagnostics). According to the user manual, the analysis was performed on the Leica Biosystems’ BOND RX Research Advanced Staining System (Doc. No. 322100-USM). The Ubiquitin C a constitutively expressed endogenous gene was used as a positive control to assess the presence of adequate RNA quality and avoid a false negative result. The dapB test was used as a negative control to assess non-specific staining, comparing the cases with negative or weakly stained SARS-CoV-2 RNA staining. Two independent authors, blinded to the characteristics of the enrolled study population, evaluated the slides. A SARS-CoV-2 RNA ISH test result was defined as positive if the cells showed brown punctate dot-like positivity. We stained the paraffin sections with antibodies against CD31, and CD68 and quantified the percentage of positive cells for specimen area (%). We performed immunohistochemistry analysis with a personal computer-based quantitative 24-bit color image analysis system (IM500; Leica Microsystem AG).

### Explanted heart study cohort and tissue sampling

Explanted heart biopsies from DM and Non-DM were also used as a control to evaluate the role of DM on ACE2 and TMPRSS2 protein independently of COVID-19. As previously described [26], from January 2010, we are conducting a prospective ongoing study (DCM-AHEAD study, NCT03546062) under ALCOA (Attributable, Legible, Contemporaneous, Original and Accurate) integrity protocols on patients who underwent first orthotopic heart transplantation (HTX) at the HTX referring center of Monaldi Hospital, Campania Region (Italy) [27, 28]. The study was approved by the Ethical Committee (prot. 438), and patients gave written informed consent. The authors divided the study population into two main groups according to whether patients did or did not have pre-transplantation DM according to American Diabetes Association (ADA) guidelines [29]. We included the patients with DM for at least 6 months before HTX, with optimal glycaemic control (HbA1c < 7.0), without the severe secondary end-organ disease (retinopathy, neuropathy, or nephropathy). This because there are strictly controlled roles for the admission on the waiting list for HTX, according to ISHLT guidelines [30]. The study was based on biopsies obtained by explanted hearts. Thus, an experienced thoracic surgeon excised 4–6 tissue specimens of about 5–10 mm^3^ from the left ventricular free wall and the explanted failing heart immediately after explant. We evaluated two tissue specimens taken from the left ventricle and either snap-frozen in liquid nitrogen or fixed in formalin for subsequent analysis. T

### Confocal laser scanning microscopy

Authors evaluated the immunofluorescence detection of ACE2 in deparaffinised biopsies of heart sections from DM and Non-DM patients affected by SARS-CoV-2 infection. Heart biopsies from DM and Non-DM subjects without COVID-19 were also analyzed as controls. Briefly, antigen retrieval buffer (10 mM Sodium citrate, 0.05% Tween 20, pH 6.0) was added at deparaffinized and rehydrated sections and boiled in the microwave for 20 min. Slides were then washed in phosphate-buffered saline (PBS) following by incubation for 30 min in Tris-buffered saline (TBS) containing 50 mM ammonium chloride to reduce background fluorescence. All sections were blocked for 1 h at room temperature (RT) in foetal bovine serum (FBS) with saponin (0.1 g/mL) and stained with primary antibodies for ACE2 (ab87436, Abcam, catalog no. 87436; 1:500) and Cardiac Troponin T [1C11] (ab87436, Abcam, catalos no. 87436; 1:500) for 16 h. Sections, incubated using Alexa Fluor 488 or 633 secondary antibodies diluted at 1:1000 in blocking solution for 1 h at R.T., were then quenched for autofluorescence using the Vector TrueVIEW Autofluorescence Quenching Kit (VEC-SP-8500, Vector Laboratories, catalog no. VEC-SP-8500-15). To ensure that what appears to be specific staining was not caused by non-specific interactions of immunoglobulin molecules with the sample, sections from DM and Non-DM patients were incubated with blocking solution. These sections were supplemented with a non-immune immunoglobulin IgG antibody, following by a secondary antibody (anti-mouse [Alexa Fluor 488 catalog no A32723, 1:500], or anti-rabbit [Alexa Fluor 633, catalog no. A-21070, 1:500] incubation for 1 h at RT. All samples were stained with DAPI (4′,6-diamidino-2-phenylindole; 5 µg/mL) for 10 min before mounting in Vectashield Mounting Medium (Vector Laboratories, catalog no. H-1700). All slides were imaged using a Zeiss LSM 710 confocal microscope with a plan apochromat X63 (NA1.4) oil immersion objective. We converted each immunofluorescence image’s individual channels into 8-bit greyscale images with a range of 0 = black to 255 = white. Within this range, the arbitrary fluorescence intensity was defined. The non-specific fluorescence signal obtained by negative control was subtracted as background.

### Protein extraction and immunoblot analysis

Explanted heart samples from DM and Non-DM patients with SARS-CoV-2 infection were used to evaluate the endogenous levels of glycosylated ACE2 and TMPRSS2 protein.

For preparation of myocardial protein extracts, 400 mL of 2D lysis buffer (7 mol/L urea, 2 mol/L thiourea, 4% CHAPS [3-([3-cholamidopropyl] dimethylammonium)-1-propane sulfonate] buffer, 30 mmol/L Tris–HCl, pH 8.8), were added to tissues (200 mg) cut into small pieces. Tissue homogenized with a Precellys 24 system (Bertin Technologies) was centrifuged at 800×*g* for 10 min at 4 °C to collect the supernatant. We precipitated the proteins by adding 100% cold methanol. 50–60 μg of sample proteins were separated by sodium dodecyl sulfate–polyacrylamide gel electrophoresis (SDS-PAGE) and then transferred to nitrocellulose membranes. Membranes were incubated for 1 h at R.T. with blocking buffer solution, TBS-T containing 20 mM Tris, pH 7.6, 100 nM NaCl, 0.1% Tween-20, and 5% non-fat dry milk under gentle shaker. Then we incubated the membranes with specific primary antibodies against ACE2 (#4355, Cell Signaling, catalog no. 4355; 1:1000) and anti-TMPRSS2 (ab109131, Abcam, catalog no. 109131; 1:2000) at 4 °C overnight, followed by incubation with peroxidase-conjugated secondary antibodies for 1 h at R.T. α-tubulin (#2125, Cell Signaling, catalog no. 2125; 1:5000) and GAPDH (ab9485, Abcam, catalog no. 9485; 1:5000) were used for protein expression normalization. We acquired the images by using Image Lab 5.2.1, Molecular Imager ChemiDoc XRS Imaging system (Bio-Rad Laboratories). Then we measured the band densities by ImageJ software (National Institutes of Health, Bethesda, USA) and expressed as arbitrary units (A.U.).

### Glycation of hACE2 and binding with SARS-CoV-2 spike protein

In vitro hACE2 glycation and binding experiments of hACE2-SARS-CoV-2 spike protein were performed by Biogem S.c.ar.l., Medicinal Investigational Research—MIR (study code: MIR 029/20). We stored the study plan and amendments, raw data, final report, and other documents pertinent to the study in the Archives of Biogem S.c.ar.l., Medicinal Investigational Research—MIR. Aliquots of hACE2 protein (residues 18 to 740, ab151852, Abcam, catalog no. 151852) were incubated in 20 mM phosphate buffer, pH 7, for 12 days at 37 °C, in the presence of 12, 60 or 120 mM of glucose. 2 Zeba Spin cartridges in 20 mM phosphate buffer, pH 7.0, was used as buffer exchange for hACE2. We treated the different aliquots of the NIST MAB 8671 in parallel as the glycation control. hACE2 glycation levels were determined by liquid chromatography/tandem mass spectrometry (LC/MS). Briefly, samples from the different glucose incubation batches were subjected to high-resolution mass spectrometry after enzymatic removal of N-linked oligosaccharides using an Ultimate 3000 HPLC (Thermo) interfaced with a Q Exactive high-resolution mass spectrometer (Thermo). The analytical protocol included tryptic digestion (8 M Guanidinium hydrochloride solution; 6 M Guanidinium hydrochloride—100 mM Tris–HCl, pH 8; 0.1 M Dithiothreitol in 20 mM Tris–HCl, pH 8; 0.28 M Iodoacetamide in water; sequencing grade trypsin 0.33 mg/mL [Promega, catalog no. V5111]; 0.1% trifluoroacetic acid in water) for 3 h at 37 °C, followed by LC/MS. A buffer exchange on Zeba Spin column equilibrated with 25 mM Tris, pH 7.5, was used for M.S. sample preparation. HPLC conditions were: column Phenomenex Kinetex XB C18, 1 × 100 mm, 2.6 mm packing material (catalog no. 00D-4496-AN) with 0.1% formic acid or 0.1% formic acid in acetonitrile and 0.2 mL/min flow rate. MS conditions were: positive polarity; sheath gas flow rate: 40; aux gas: 8; sweep gas: 0; spray voltage: 3.3. KV; capillary Temperature: 320 °C; s-Lens R.F. Level: 50. Full MS settings were resolution: 35,000, AGC Target: 1e6; maximum injection time: 100 ms; isolation window: 4 *m*/*z*; scan range: 200 to 2000 *m*/*z*. MS/MS settings were: exclude isotopes: on; dynamic exclusion: 20 s. Additionally, the glycosylation associated structural heterogeneity of hACE2 was removed by overnight incubation at 37 °C with PNGase F (Promega, catalog no. V4831), and then evaluated the result by SDS-PAGE. NIST8671 monoclonal antibody was glycosylated in the same conditions as hACE2, as glycation control. We used a dedicated bioinformatics approach based on a custom-made computer script that guided the identification of glycated lysine residues (Algorithm logics of computer-based prediction of glycated peptides written in the Ruby programming language). We calculated the glycation percent as the weighted peak area percent of the glycated peptide over that of non-glycated counterparts (sum of completely and partially digested fragments) L.C. conditions. We performed the affinity between hACE2, at different glycation stages, and SARS-CoV-2 Spike protein (catalog no. RP9720110150, BioVendor R&D) by Surface Plasmon Resonance (SPR), Biacore T200 (G.E. Healthcare). SARS-CoV-2 Spike protein (dimer) was immobilized using carboxymethyl dextran-coated CM5 sensor chips using standard amine coupling chemistry. We used up to 20 µg of SARS-CoV-2 Spike protein in each immobilization reaction. We performed the set-up experiments to establish the amount of ACE2 and the optimal pH for the immobilization phase. Biacore analysis samples were prepared using the buffer exchange on Zeba spin column (Desalting columns, 7K MWCO, p/n 89882) and equilibrated with Biacore buffer (Cytiva, catalog no. BR-1006-09). Spike-S1 protein tagged with Human IgG Fc was diluted in HBS-EP plus running buffer at different concentrations, ranging from 2 to 1000 nM. 1 µg/mL of protein was immobilized and injected for 180 s at a flow rate of 10 µL/min for antibody capture. Approximately 20 µg of protein per run was required. For each assay condition (presence/absence of glucose), every ACE2 sample was run at least four different concentrations (64, 32, 16, 8 and 4 µg/mL) on the sensor-bound Spike protein and injected for 120 s at a flow rate of 30 µL/min. Formulation buffer was run as a control. Dissociation was followed for 300 s, regeneration was achieved with a 60-s pulse of 3 M MgCl_2_. Ligand-analyte affinity (K.D.) and kinetic parameters (association rate, K_a_; dissociation rate, K_d_) of Originator and Biosimilar Rituximab were calculated with the Biacore T200 Evaluation Software (version 2.0; G.E. Healthcare).

#### Data and resource availability

This information is available if required.

### Sample size calculation and data collection

For this study, we calculated a sample size of 35 participants for each group, with estimated 80% power to detect a change of 0.015 between the study endpoints of the DM and non-DM groups 5% level of significance. To date, we assumed a 20% loss due to early withdrawals and non-evaluable measurements and, combined with the effect of stratification on analysis, resulted in the requirement to recruit at least 30 patients per treatment group.

### Statistical analysis

Data were presented as mean ± SD. Continuous variables were compared among the groups of patients with Student’s t-test or one-way ANOVA for normally distributed data and Kruskal–Wallis for non-normally distributed data. When differences were found among the groups, Bonferroni correction was used to make pairwise comparisons. Only p values of 0.05 or lower were considered statistically significant. When representative images were shown, the selected images were those that most accurately represented the average data obtained in all the samples. All calculations were performed using SPSS 23 software (SPSS Inc, Chicago, IL, USA).

## Results

### Clinical characteristics and cardiomyocytes SARS-CoV-2RNA on COVID-19 autopsy cohort and in non-COVID-19 explanted heart cohort

#### COVID-19 autopsy cohort

**Fig. 1:**
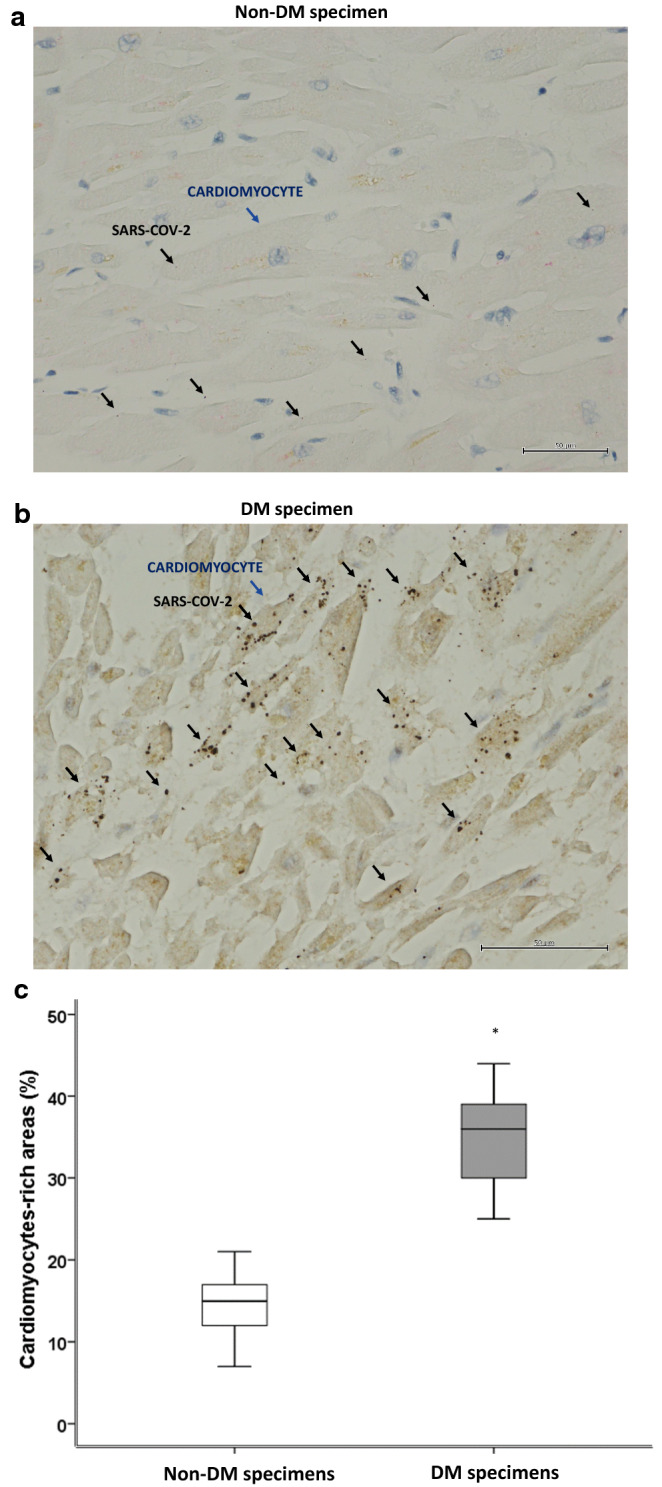
SAR-COV-2 in myocardial tissue from COVID-19 autopsies.** a** Representative myocardial tissue specimen from 60 patients without diabetes (Non-DM) (× 400). **b** Representative myocardial tissue specimens from 37 patients with diabetes (DM). Brown punctate evidenced the SARS-COV-2 RNA copies in the cardiomyocytes (96 positive cells/237 cells) (× 400). These structures are marked with black arrows (SARS-COV2), and with blue arrows (Cardiomyocytes). **c** Mean ± SD of the percentage of SARS-COV-2 positive cardiomyocyte. Statistical test: Student’s t-test. Bonferroni correction was used to make pairwise comparisons. *P < 0.05

The median (interquartile range) age of the 97 individuals was 63 (69–75) years, and 60 (612%) were male. None of the patients in the study were diagnosed as having clinically fulminant myocarditis. All patients were given the full COVID-19 therapy (Table 1). Authors diagnosed D.M. in 37 patients (38%) (Table 1). Duration of hospitalization and BMI were significantly higher in DM patients (P = 0.002, P = 0.019, respectively). In situ hybridization of SARS-CoV-2 RNA evidenced the virus presence in myocardial tissue (Fig. 1). The most frequent localization of SARS-CoV-2 in DM myocardial tissue was in the cardiomyocytes (Fig. 1). Fourth-seven of 97 autopsies (48%) had SARS-CoV-2RNA in cardiomyocytes. Thirty of 37 DM autopsy cases (81%) and 17 of 60 Non-DM autopsy cases (28%) had SARS-CoV-2RNA in cardiomyocytes (Fig. 1). The DM vs. Non-DM specimens had a significantly higher number of cardiomyocytes with SARS-COV-2 localization (34.7 ± 5.3% vs. 14.3 ± 4.1%, P = 0.001), (Fig. 1).

**Table 1:** Clinical characteristics of COVID-19 autopsy cohort

	Diabetic patients (N = 37)	Non-diabetic patients (N = 60)	P
Age, years	65.9 ± 10.9	69.3 ± 9.4	0.107
Male, n (%)	23 (62.2)	37 (61.7)	0.515
BMI, kg/m^2^	28.9 ± 6.4	26.2 ± 1.9	0.019
Duration symptoms, days	14.3 ± 1.9	15.1 ± 2.1	0.065
Duration Hospitalization, days	11.3 ± 1.1	9.7 ± 1.2	0.002
Dyslipidemia, n (%)	7 (15.2)	30 (23.7)	0.181
Hypertension, n (%)	23 (62.2)	36 (60.0)	0.502
Obesity, n (%)	15 (40.5)	20 (33.3)	0.307
Cardiovascular disease, n (%)	12 (32.4)	21 (35.0)	0.322
COPD, n (%)	22 (59.5)	34 (56.7)	0.478
Smoking, n (%)	8 (21.6)	12 (20.0)	0.522
Patients with SARS-COV-2 infected cardiomyocytes, n (%)	30 (81.1)	17 (28.3)	0.001
Patients with SARS-COV-2 infected endothelial cells, n (%)	10 (27.0)	22 (36.7)	0.225
Patients with SARS-COV-2 infected macrophages, n (%)	20 (54.1)	41 (68.3)	0.116
Covid-19 drug therapy			
Antiviral (%)	37 (100)	60 (100)	/
Antibiotics (%)	32 (86.5)	51 (85)	0.396
Chinidine (%)	30 (81.1)	50 (83.3)	0.512
Glucocorticoids (%)	29 (78.4)	49 (81.7)	0.215
Tocilizumab (%)	4 (10.8)	6 (10)	0.510
Oxygen inhalation (%)	31 (83.8)	50 (83.3)	0.256

Data are means ± SD or n (%)

*BMI* body mass index, *COPD* chronic obstructive pulmonary disease, *SARS-COV-2* severe acute respiratory syndrome coronavirus 2

#### Non-COVID-19 explanted heart cohort

Explanted hearts were divided into two groups: Non-DM patients (n = 93, 69%) and DM patients (n = 47, 34%). DM patients were more likely to have CHD as a reason for HTX (Table 2). None of the other baseline characteristics differed significantly between groups, most notably the preoperative levels of creatinine and cholesterol. Anti-DM therapy of patients with pre-transplant DM was reported in Table 1. DM duration was 14.8 ± 2.8 years. None of the DM HTX had complications such as nephropathy, neuropathy, or retinopathy.

**Table 2:** Clinical characteristics of non-COVID-19 explanted heart cohort

	DM patients	Non-DM patients	P
N	47	93	/
Mean age (years)	52.9±6.7	53.7±4.1	0.19
Sex, male (%)	35 (74)	73 (78)	0.36
BMI (kg/m^2^)	27.3±1.2	25.7±1.6	0.001
Aetiology of heart failure, n (%)			
Ischemic cardiomyopathy	25 (53)	50 (54)	0.54
Dilated cardiomyopathy	20 (42)	37 (40)	0.27
Other	2 (4)	6 (6)	0.33
Cardiovascular risk factors, n (%)			
Hypertension, n (%)	12 (25)	25 (27)	0.52
Dyslipidemia, n (%)	21 (45)	30 (32)	0.10
Family history of CAD, n (%)	27 (57)	45 (48)	0.20
Smoking history, n (%)	5 (11)	10 (11)	0.61
Laboratory analyses			
Plasma glucose (mg/dl)	126.7±18.7	88.7±6.7	0.001
HbA1c (%)	6.7±1.2	4.8±0.8	0.007
Cholesterol (mg/dl)	177.1±21.6	161.7±18.7	0.011
LDL-cholesterol (mg/dl)	101.1±22.7	97.4±16.7	0.16
HDL-cholesterol (mg/dl)	40.9±1.1	41.8±1.7	0.21
Triglycerides (mg/dl)	172.4±28.2	113.4±11.1	0.013
Creatinine (mg/dl)	1.0±0.37	1.0±0.26	0.74
Therapy			
ACEi, n (%)	40 (85)	83 (89)	0.33
ARBs, n (%)	15 (32)	34 (37)	0.36
Diuretic, n (%)	45 (96)	92 (99)	0.26
Antiaggregant, n (%)	46 (98)	83 (89)	0.06
Anticoagulant, n (%)	5 (11)	16 (17)	0.22
Statin, n (%)	40 (85)	70 (75)	0.12
Beta-blockers, n (%)	47 (100)	90 (97)	0.55
Sacubitril-valsartan, n (%)	21 (45)	31 (33)	0.13
Nitrate, n (%)	21 (45)	37 (40)	0.35
Calcium-antagonist, n (%)	7 (15)	6 (6)	0.96

Data are means ± SD or n (%).

*BMI* body mass index, *DM* diabetes mellitus, *Non-DM* without diabetes mellitus, *HbA1c* glycated haemoglobin, *LDL* low-density lipoprotein, *HDL* high-density lipoprotein, *ACEi* angiotensin-converting enzyme inhibitors, *ARBs* angiotensin II receptor blockers

### Myocardial ACE2 and TMPRSS2 expression in COVID-19 autopsy cases and non-COVID-19 explanted hearts

**Fig. 2:**
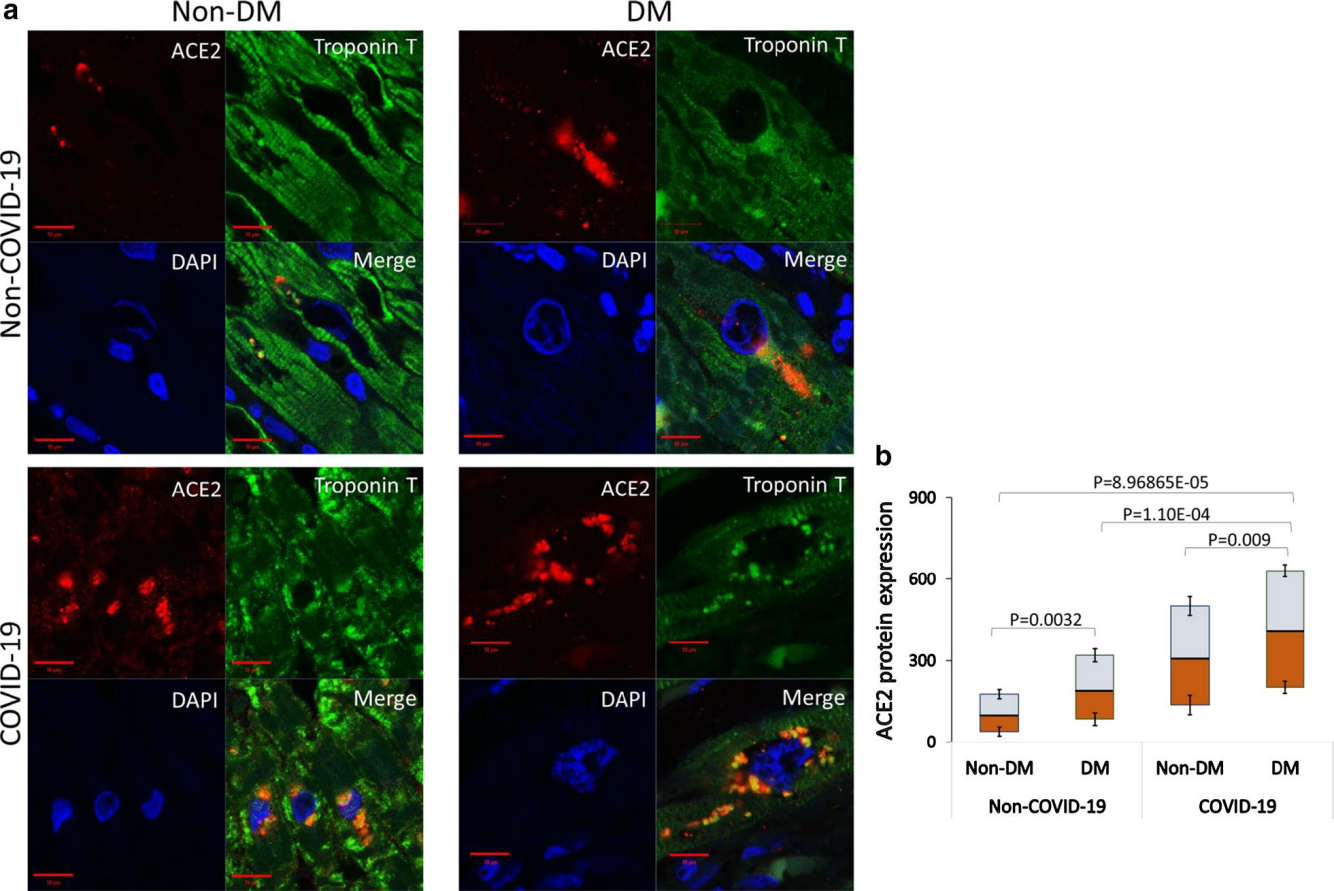
ACE2 immunofluorescence detection. **a** Representative images of ACE2 expression (red) and cardiac troponin T (green) in non-COVID-19 and COVID-19 heart tissue from patients with diabetes (DM) and patients without diabetes (Non-DM). Cell nuclei were stained blue with DAPI. **b** Fluorescence intensity analysis in the Non-DM Non-COVID-19 (n = 43) versus DM Non-COVID-19 (n = 7), (p = 0.0032) and Non-DM COVID-19 (n = 17) versus DM COVID-19 (n = 30), (p = 0.009) of myocardial ACE2 expression was estimated with Image J software. Analysis comparing DM COVID-19 versus Non-DM Non-COVID-19 (p = 8.96865E−05) and DM COVID-19 versus DM Non-COVID-19 (p = 1.10E−04) was also reported. Shown as mean ± SD. Statistical test: Student’s t-test. Bonferroni correction was used to make pairwise comparisons. Data were presented as box and whisker plots showing medians (middle line) and in boxes the third and first quartiles (75th and 25th percentiles), while the whiskers show 1.5 × the interquartile range (IQR) above and below the box. Scale bar = 10 µm. *ACE2* angiotensin-converting enzyme 2

**Fig. 3:**
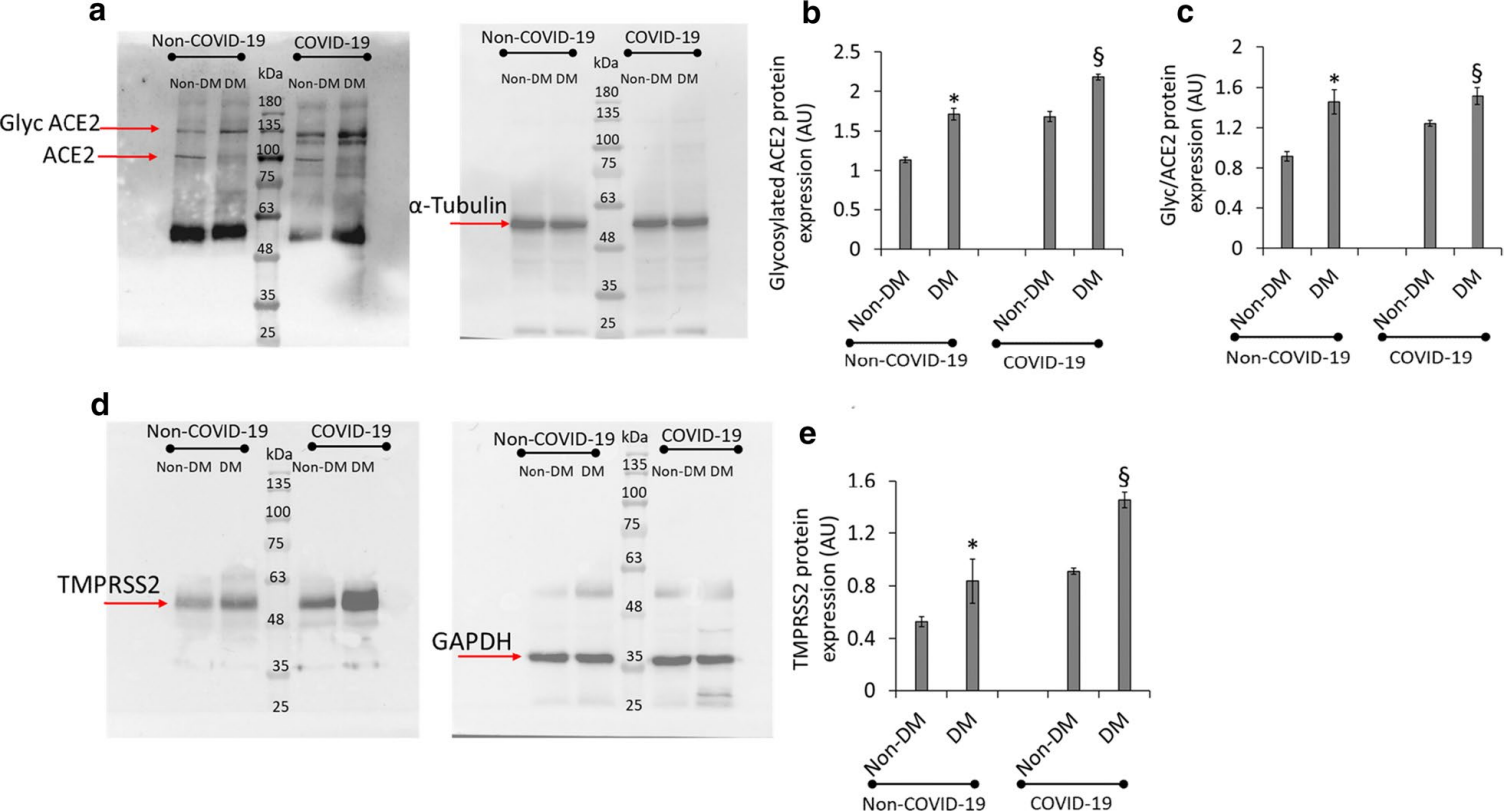
Glycosylated ACE2 and TMPRSS2 protein levels. **a–c**, Representative images and bar graph of Western blotting analysis (n = 4) of glycosylated (Glyc) ACE2, total ACE2 and Glyc/total ACE2 ratio in heart tissue from patients without diabetes (Non-DM) Non-COVID-19 (n = 43) versus heart tissue from patients with diabetes (DM) Non-COVID-19 (n = 7), (p = 0.03276 and p = 0.047, respectively) and Non-DM COVID-19 (n = 17) versus DM COVID-19 (n = 30), (p = 0.002391 and p = 0.0025, respectively). Shown as mean ± SD. Statistical test: Student’s t-test. Bonferroni correction was used to make pairwise comparisons. **d**, **e** Representative images and bar graph of Western blotting analysis (n = 3) of TMPRSS2 in Non-DM Non-COVID-19 (n = 43) versus DM Non-COVID-19 (n = 7), (p = 0.0344) and Non-DM COVID-19 (n = 17) versus DM COVID-19 (n = 30), (p = 0.001) heart samples. Protein expression was determined by ImageJ 1.52n software and quantified using α-tubulin or GAPDH. Values are expressed as arbitrary units (A.U.). Shown as mean ± SD. Statistical test: See (**a**–**c**). *p < 0.05 vs. non-DM (Non-COVID-19); ^§^p < 0.01 vs. non-DM (COVID-19). *ACE2* angiotensin-converting enzyme 2, *TMPRSS2* transmembrane protease serine 2, *GAPDH* glyceraldehyde 3-phosphate dehydrogenase

Immunofluorescence analysis of myocardial ACE2 expression levels in heart sections from DM and Non-DM cases showed a consistent increase of myocardial ACE2 protein expression in DM patients compared to Non-DM subjects (p < 0.0032). Myocardial ACE2 expression of COVID-19 patients with DM also was upregulated compared to Non-DM patients (p < 0.009) (Fig. 2). Notably, the ACE2 detection at cardiomyocyte level, provided by a cardiac troponin T double-staining, showed a peri-nuclear ACE2 localization mainly in DM sections (Fig. 2). Negative control sections from DM and Non-DM patients were reported in Additional file 1: Figure SI. Given the inability to discriminate between the total and glycosylated (Glyc) form of ACE2 by immunofluorescence analysis, we performed Western blot assay using a specific ACE2 antibody which, while still binding to the non-glycated form (detected band at ≈ 90 kDa), recognizes endogenous level of glycosylated ACE2 protein (Fig. 3a). Results showed a higher glycosylated ACE2 content (detected band at 135 kDa) in the heart from DM compared with Non-DM patients (p < 0.05) (Fig. 3b). The increase of glycosylated ACE2 was even higher in COVID-19 patients with DM as compared to COVID-19 Non-DM patients (p < 0.01) (Fig. 3b). The ratio between glycosylated and total ACE2, confirmed the higher glycosylated protein content (p < 0.05) in DM patients for each condition (Fig. 3c). The effect of DM on the expression of myocardial TMPRSS2 protein levels was next investigated in the explanted heart from non-COVID-19 failing heart DM and Non-DM patients. We found an increase of glycosylated ACE2 and TMPRSS2 protein content in DM vs. Non-DM specimens (p < 0.05) (Fig. 3d). COVID-19 myocardial tissue from DM patients showed a more pronounced TMPRSS2 protein expression level compared to Non-DM (p < 0.01) (Fig. 3d, e).

### hACE2 glycation and binding to SARS-CoV-2 Spike

**Fig. 4:**
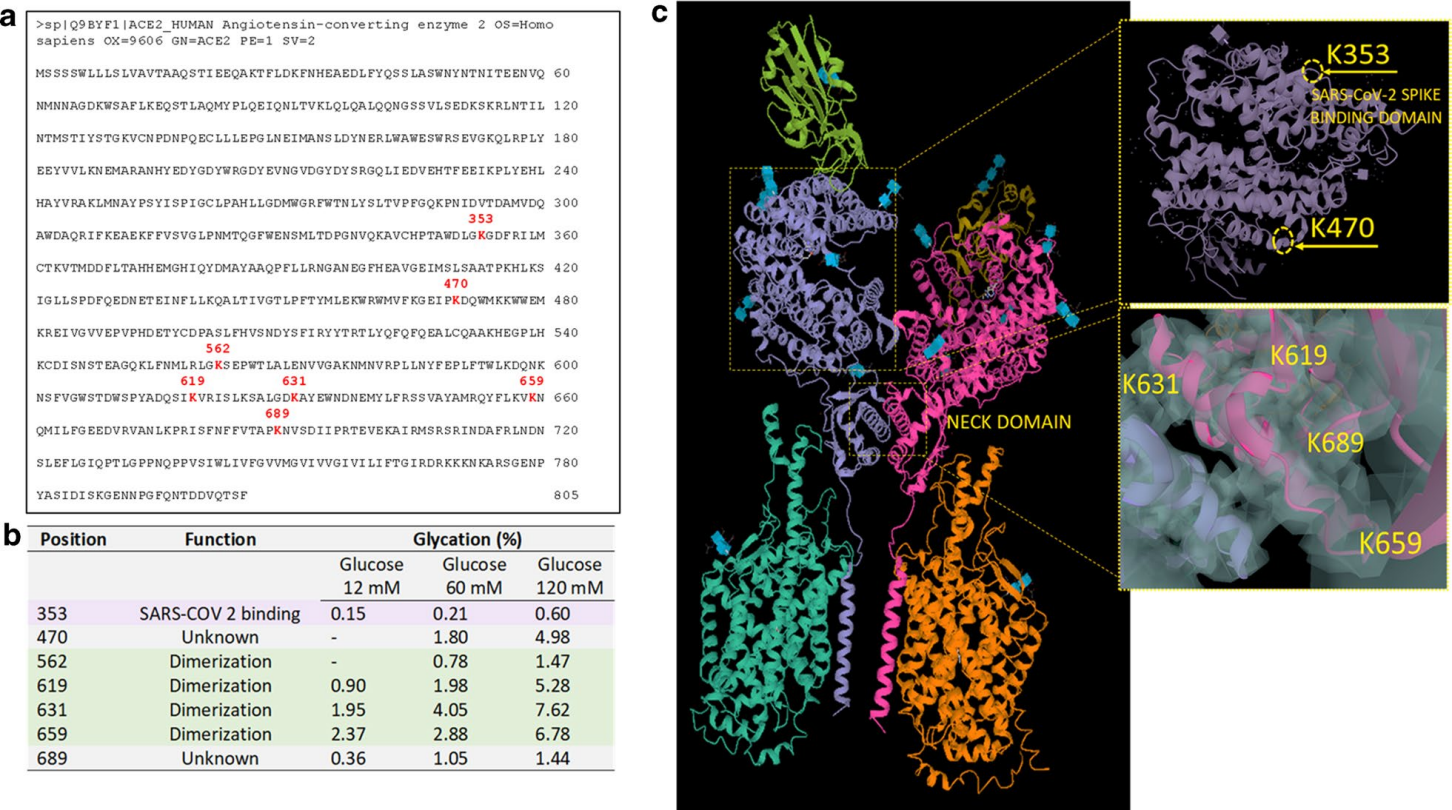
Mapping glycation on hACE2.** a** Human ACE2 sequence (www.uniprot.org; entry: Q9BYF1, entry name: ACE2_HUMAN) showing in red the glycated lysine residues obtained after 12 days of incubation with 120 mM of glucose. **b** Position glycated lysine (K) after 12 days of incubation with 12 mM, 60 mM, and 120 mM of glucose and function of glycated sites. **c** Human ACE2 homodimer (PDB 1r42) showing the lysine 353 (K353), involved in the Spike-RBD binding to ACE2, lysine 470 (K470) (unknown function). ACE2 structure from PDB 6M17 showing the glycated lysine 619 (K619), 631 (K631), 659 (K659), and 689 (K689) in the polar neck region involved in the dimerization of ACE2

Tryptic digestion followed by LC/MS experiments allowed the identification of every glycation event that increased the target protein mass of 162 kDa and their relative glycation levels (Fig. 4). Glycated lysine residues were unambiguously identified from a proper pattern composed of mass, electric charge, and retention time properties, and seven glycation hot spots of hACE2 were found, and their mass spectra were reported in Data Supplement (Additional file 1: Figure SII–SVIII). They all showed high-quality isotope envelopes, expected mass signature of glycation, high mass accuracy, and expected chromatographic behavior. All these criteria fitted together were used for the unambiguous identification and also for a quantitation based on intensity relationships of glycated and non-glycated signals. All hACE samples were found to have the same lysine residues involved in the in vitro glycation (Fig. 4a) even though they could not be predicted in advance since no known structural consensus (either sequence or 3D). Also, the level of glycation showed a direct (although not linear) relationship between the incubation media's glucose concentration (Fig. 4a). The percentage of glycation was calculated as the weighted peak area percent of the glycated peptide over non-glycated counterparts (sum of completely and partially digested fragments). Results showed that glycation was detected after 12 days of incubation with glucose for all three concentrations tested (12, 60, and 120 mM). Seven glycation sites were detected from over forty predicted potential sites. These glycation sites were maintained in all analyzed samples, with glycation rate showing a dose-dependency on the glucose concentration (Fig. 4a). The glycated lysine residues are shown in red on the hACE2 sequence reported in the Uniprot database (www.uniprot.org; entry: Q9BYF1, entry name: ACE2_HUMAN) (Fig. 4b). Identification of glycation sites was based on mass analysis and retention time data with very high accuracy for all samples tested (Additional file 1: Table SI). Finally, all retention time patterns (glycated peptides eluted very close to their non-glycated counterparts, often in slight advance) were found to be very reproducible, supporting the unambiguous identification just described.

Results showed that among the seven glycated residues, only lysine 353 (Lys 353) (Fig. 4c) has previously been reported as important for binding Spike-RBD [31]. However, under our experimental conditions, minor glycation of Lys 353 (0.6%) was found. Also, in the hACE2 (Protein Data Bank, PDB 1r42), the Lys 470 with unknown function displayed a 4.98% of glycation at 120 mM of glucose (Fig. 4c). Notably, a higher number of glycated residues were detected in the polar region of ACE2 involved in the dimerization (neck domain), with Lys 619, Lys 631, Lys 659, and Lys 689 showing 1.47%, 5.28%, 7.62%, and 6.78% of glycation, respectively (Fig. 4c). The structures shown in Fig. 4c are from PDB 6M17. Next, we verified that the glycosylation-associated structural heterogeneity of hACE2 was quite difficult to eliminate. ACE2 glycosylation was removed by overnight incubation with PNGase F. SDS-PAGE results showed that the deglycosylated hACE2 has a lower apparent molecular weight due to the PNGase digestion. However, its band is still heterogeneous with a diffused aspect (Additional file 1: Figure SIX).

For SPR measurements of glycated ACE-SARS-CoV-2 Spike protein binding, an affinity coupling approach using a biosensor functionalized with an anti-human F.C. antibody immobilization kit (Cytiva, catalog no. BR-1008-39) was used. This system allowed to bind the Spike protein via its F.C. affinity tail with chip recycling for the whole study. The system showed to be functional, and an amount of 1 µg/assay of immobilized Spike protein was found to work optimally. In line with the low grade of glycation found at the Lys 353 located in the hACE2 domain of binding for SARS-CoV-2 Spike, results showed a minimal influence of glycation on the binding properties to SARS-CoV-2 Spike, as indicated by the K_a_ and K_d_ and the protein ligand-analyte affinity (K.D.) (9.69 nM in control and 11.07 nM in glycated hACE2 with 120 mM of glucose) (Additional file 1: Figure SX).

### Glycation modifies ACE migration

**Fig. 5:**
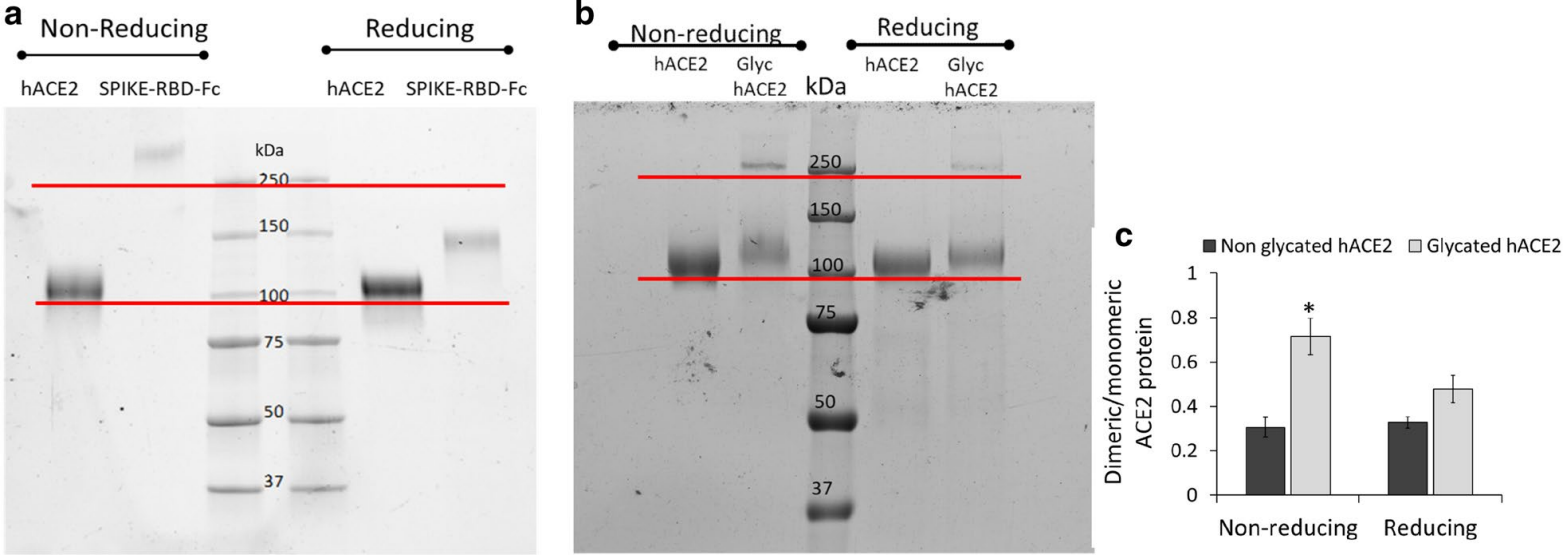
Effect of glycation on hACE2 migration.** a** SDS-PAGE was conducted using hACE2 and SARS-CoCOVV-2 Spike protein aliquots collected before starting glycation and SPR measurements. **b** SDS-PAGE was conducted using 8% gels in reducing and non-reducing conditions with hACE2 incubated for 12 days with glucose 120 mM (Glyc hACE2). Molecular weight indicators are displayed at the center. **c** Ratio of the dimeric to monomeric form of ACE2 in reducing and non-reducing condition. *p < 0.05 vs. non glycated ACE2. Shown as mean ± SD. Statistical test: Student’s t-test

The impact of the glycated residues detected in the neck domain of ACE2 on the protein structure was further investigated by SDS-PAGE under reducing and non-reducing conditions. To this end, the oligomerization state of hACE2 and SARS-CoV-2 Spike protein was first evaluated in samples before starting mild glycation experiments and binding measurement, confirming the purity of dimeric spike and the monomeric hACE2 protein (detected band at 100 kDa) used in this study (Fig. 5a). Notably, the SDS-PAGE of glycated hACE2 (Glyc-hACE) (120 mM glucose) showed important differences compared to non-glycated hACE2 (hACE) (Fig. 5b). In a non-reducing setting, hACE showed only one band at 100 kDa, whereas the Glyc-hACE showed both a predominant band over 100 kDa along with another band at the higher molecular weight (250 kDa). Under reducing conditions, both hACE and Glyc-hACE showed the predominant band of at 100 kDa. However, despite the experimental reducing conditions, a weak band at 250 kDa was still observed for Glyc-hACE. The evidence of glycation role on ACE2 dimer formation under a non-reducing setting (p < 0.05) was provided by the quantitative ratio of the dimeric to monomeric form (Fig. 5c).

These results indicated the mild glycation modified hACE2 protein migration and oligomerization process, supporting non-enzymatic glycation at neck domain level on hACE2 oligomerization and dimer formation (Addition file 2).

## Discussion

For the first time in literature, our investigation provides a new clinical human and an “in vitro-ex vivo” post-mortem model to evaluate the SARS-COV-2 ligand with ACE2 receptor, its entrance, and replication in cardiomyocytes in patients with normoglycemia, and under a condition of over-glycaemic stress. Notably, in the COVID-19 autopsy cohort, the DM vs. Non-DM specimens had a significantly higher number of SARS-COV-2 localized inside the cardiomyocytes. Thus, we evidenced, by immunofluorescence analysis of cardiac specimens, a higher expression of myocardial ACE2 protein in DM vs. Non-DM. Notably, DM sections mainly evidenced a peri-nuclear ACE2 localization. We also observed in COVID-19 patients an increase of ACE2 in heart specimens from DM as compared to Non-DM patients. In this setting, in the immunoblotting analysis of explanted hearts from non-COVID-19 patients, we found an increase of glycosylated ACE2 and TMPRSS2 protein content in DM vs. Non-DM specimens. Notably, it was highest in COVID-19 myocardial tissue of DM vs. Non-DM. Furthermore, the ratio between glycosylated on total ACE2, confirmed the more consistent expression levels of glycosylated ACE2 protein in COVID-19 patients, with the highest upregulation in DM vs. Non-DM (Additional file 3).

From current literature, authors reported that SARS-CoV-2 could infect human cardiomyocytes in culture and different models of cardiac tissue [31–33]. On the other hand, these authors did not provide any evidence about cardiomyocyte SARS-CoV-2 infection in the subgroup of high-risk patients, such as DM patients. Secondly, they did not assess the specific pathways induced by DM to promote the virus entry into the cardiomyocyte. In this regard, the heart shows a higher expression of the ACE2 receptor, which could mediate SARS-CoV-2 cell entry [34]. Notably, during SARS-COV-2 infection, the trimeric Spike protein is cleaved into S1 and S2 subunits with the S1 containing the RBD that directly binds to the peptidase domain ACE2 [34]. The binding kinetics between the SARS-CoV-2 spike protein and the ACE2 receptor depends on protein structures, and their molecular interactions are altered by glycosylation [35–37]. In particular, in the SARS-CoV-2/RBD–ACE2 complex structure, the glycan–RBD interaction has important roles in the binding [37]. Indeed, it is a chain of Asn90-linked NAG–NAG–β-d-mannose in contact with the Thr402 of the SARS-CoV-2/RBD [38]. In this context, we observed a consistent increase of ACE2 protein expression in heart tissue of DM vs. Non-DM patients (p < 0.0032). However, to elucidate the supposed model of intra-cellular entrance and replication of SARS-COV-2, in the in vitro model, we evaluated the ACE2 glycation and binding to SARS-CoV-2 Spike. Thus, we detected the ACE2 glycation after 12 days of incubation with glucose for all three concentrations tested (12, 60, and 120 mM). Notably, despite the minimal influence of glycation on the binding properties to SARS-CoV-2 Spike, we evidenced the effect of chemical modification on the ACE2 oligomerization state. However, the mild glycation modified ACE2 protein migration and oligomerization process could support non-enzymatic glycation at neck domain level on hACE2 oligomerization and dimer formation. Furthermore, there is a higher expression of glycosylated ACE2 in heart samples from DM vs. Non-DM patients (p < 0.05), with more consistent levels in COVID-19 patients with DM (p < 0.01). To date, this result could support the existing relationship between DM and the upregulation of glycosylated ACE2 protein. Again, COVID-19 patients with DM also showed higher levels of TMPRSS2 expression. This study result could confirm the hypothesis that the glycosylation of ACE2 could be linked to a higher expression of TMPRSS2 protein [39].

Therefore, in DM vs. Non-DM patients, the upregulation of ACE2 expression (total and glycosylated forms) in cardiomyocytes, along with non-enzymatic glycation, could increase the susceptibility to COVID-19 infection in DM patients by favoring the cellular entry of SARS-CoV2. In this context, the endothelial and platelet cells pathology, and fibrin(ogen) over-synthesis could negatively interact with SARS-CoV-2 in DM vs. Non-DM patients. Indeed, in DM patients there is a higher rate of clotting in COVID-19 patients [8], that is linked to a prominent elevation of fibrinogen and d-dimer/fibrin(ogen) degradation products, causing a hypercoagulability [40]. Notably, the degree of d-dimer elevation positively correlated with mortality in COVID-19 patients [40]. In addition to arterial thrombotic events and microvascular thrombotic disorders, COVID-19 patients often have mild thrombocytopenia and appear to have increased platelet consumption, together with a corresponding increase in platelet production [41]. Conversely, if the entry of the SARS-COV-2 into the host cell requires the cleavage of the S protein into the S1 and S2 subunits by TMPRSS2 to promote pathogenicity and myocardial damage reported in COVID-19 patients [19, 20], we might speculate that DM vs. Non-DM might over-express these adverse clinical pathways. Therefore, all these pathological processes might be over-expressed in DM vs. Non-DM, and mainly induced by SARS-CoV-2 in DM vs. Non-DM patients. To date, this is in line with our study results. Indeed, we evidenced, (i) a significantly higher number of cardiomyocytes with SARS-COV-2 localization (34.7 ± 5.3% vs. 14.3 ± 4.1%, P = 0.001) in the DM vs. Non-DM specimens (Fig. 1); (ii) a consistent increase of myocardial ACE2 protein expression in DM patients compared to Non-DM subjects in both explanted hearts from COVID-19 patients and controls; (iii) a higher glycosylated ACE2 and TMPRSS2 protein content in the heart from DM compared with Non-DM patients in both explanted hearts from COVID-19 patients and controls (Fig. 3b, c); (iv) that COVID-19 myocardial tissue from DM patients had a more pronounced TMPRSS2 protein expression level compared to Non-DM (p < 0.01) (Fig. 3d, e). However, we might suggest that all these pathogenic mechanisms over-expressed in DM vs. Non-DM could promote pathogenicity and myocardial damage in controls and much more in a condition of SARS-CoV-2 infection.

Thus, here we hypothesized that increased glycosylated ACE2 and TMPSS in cardiomyocytes, due to DM milieu, might impact the SARS-COV-2 cells entry. However, this might represent a crucial pathophysiological step, leading to worse clinical outcomes in COVID-19 patients with DM.

Although it is well recognized that ACE2 glycation is linked to a DM condition, the pathways that promote the entry of SARS-COV-2 in the cells are determined by spike glycation [41]. The mechanism by which DM milieu may be involved in this process is not fully clarified. Thus, to elucidate this aspect, we conducted an in vitro study to evaluate the occurrence of non-enzymatic glycation of ACE2 and its possible pathogenic role in the changing of the protein binding to SARS-CoV-2 Spike or the protein structure in terms of the amino acid (mostly lysine) glycation. However, the evaluation of the functional impact of non-enzymatic glycation on ACE2 [37–40] showed seven glycated residues of which only one (Lys 353) in the important for the binding domain for Spike-RBD, and four (Lys 619, Lys 631, Lys 659, and Lys 689) in the neck domain involved in ACE2 dimerization [36–42]. In line with the low percentage of glycation detected at Lys 353, we found that the mild glycation of ACE2 monomer slightly influenced the binding properties to Spike protein. Then, the evaluation of the oligomerization state of ACE2 after in vitro mild non-enzymatic glycation, besides the predominant band of monomeric ACE2 (100 kDa), unveiled the occurrence of a band at 250 kDa under non-reducing conditions. This supports the occurrence of changes in the oligomerization process towards dimer formation. Indeed, under reducing conditions, the glycated ACE2 showed an increase in the band at 100 kDa and decreased in the band at 250 kDa. Although the band at 250 is detectable in our experimental conditions in small amounts, it cannot exclude that the long-term effect of diabetes on glycation could significantly influence the oligomerization favoring dimeric ACE2. This evidence could reflect the highest susceptibility of DM to SARS-CoV-2 infections because oligomerization increases the binding affinity and avidity to SARS-CoV-2 Spike protein. Indeed, as recently demonstrated, the trimeric engineered ACE2 variant has a binding affinity of ~ 60 pM for SARS-CoV-2 Spike protein, compared with 77–90 nM for monomeric ACE2 and 12–22 nM for dimeric ACE2 constructs [16, 18, 43]. Finally, although dimeric ACE2 is unlikely to engage more than one RBD from the same spike protein, it cannot be ruled out that higher levels of ACE2 dimers on the cell surface might further cluster to induce more RBDs. Then, the RBDs could adopt up conformation and help SARS-CoV-2 to transit from the pre-fusion state to the post-fusion state. However, in addition to the known adverse effects of hyperglycemia in patients with COVID-19 [43–45], here we reported the enhanced expression of glycosylated ACE2 in DM cardiomyocytes.

**Fig. 6:**
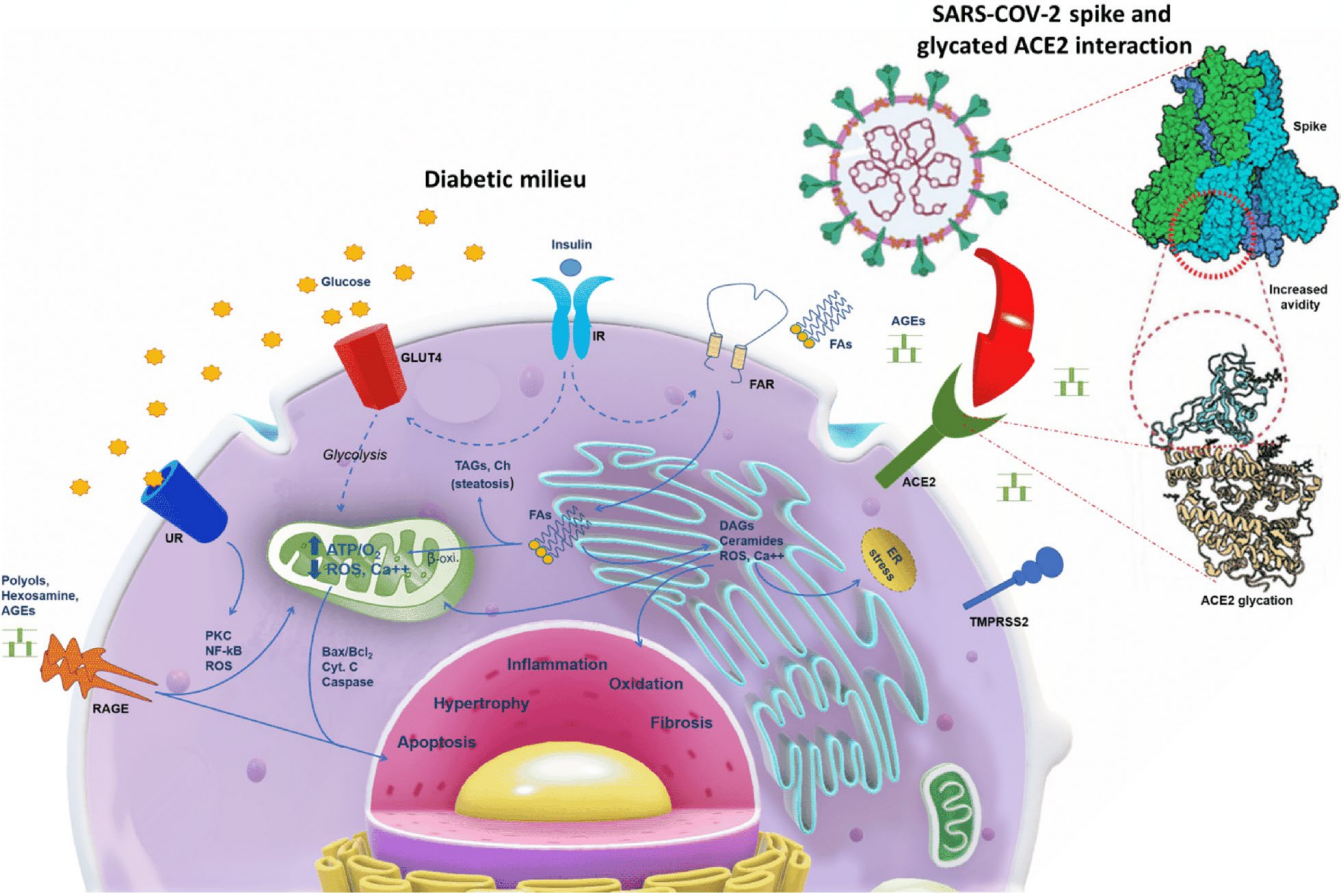
Schematic representation of the proposed effect of diabetes milieu on ACE2 in the heart of patients with type 2 diabetes. In patients with diabetes, the enhanced long-term non-enzymatic glycation of ACE2 at the neck domain of dimerization can affect ACE2 oligomerization and, consequently, its avidity for SARS-COV-2 Spike binding, potentially favoring cardiomyocyte virus entry

Furthermore, this result provides the first evidence on the in vitro glycation of ACE2 at the neck domain of dimerization. Indeed, the enhanced ACE2 glycation suggests that this system's activation by DM milieu is associated with an increased ACE2 oligomerization and avidity for SARS-COV-2 Spike binding. Finally, these effects could favor cardiomyocyte rather than interstitial or endothelial cell virus invasion (Fig. 6).

On the other hand, because of data from autopsied cases, we cannot exclude that post-mortem condition may influence the expression of ACE2 and TMPSS, such as observed in DM patients. Thus, to exclude possible post-mortem condition-induced changes, we evaluated the effects of DM milieu on ACE2 and TMPSS expression in the explanted hearts. Moreover, according to the autopsied data, we evidenced an increase of glycosylated ACE2 and TMPRSS2 protein content in DM specimens compared to Non-DM specimens from non-COVID-19 hearts (Additional file 4).

## Conclusion

Furthermore, taken together all these data might suggest that DM might promote the ACE2 modifications, favoring SARS-COV-2 entry in cardiomyocytes, independently of both post-mortem and COVID-19 molecular changes. Thus, we might speculate that increased ACE2 glycation and TMPSS expression in cardiomyocytes, as a consequence of DM, may favor SARS-COV-2 entry in the host cell. Therefore, this could represent a crucial pathophysiological step leading to worse clinical outcomes and cardiovascular events in COVID-19 patients with DM [46].

## Supplementary Information

**Additional file 1**: **Figure SI**. Immunofluorescence negative control. Sections from diabetic and non-diabetic patients were performed with blocking solution, supplemented with a non-immune immunoglobulin IgG antibody, following by secondary antibody Alexa Fluor 488 or 633 incubation for 1 hour at RT. All samples were stained with DAPI (5 μg/ml) for 10 min before mounting in Vectashield Mounting Medium (Vector Laboratories, Burlingame, CA, USA). All slides were imaged using a Zeiss LSM 710 confocal microscope (Zeiss, Oberkochen, Germany) with a plan apochromat X63 (NA1.4) oil immersion objective. DM: diabetes mellitus; Non-DM: without diabetes mellitus. **Table I**: Mass accuracy and retention time (RT) of hACE2 tryptic peptides targeted by glycation. The measurement accuracies were reported in parts per million (ppm) and the chromatographic retention times in minutes (mins). **Figure SII**: Mass spectra of the triply charged hACE2 tryptic peptide 342-357 in the glycated (panel A) and non-glycated (panel B) form. **Figure SIII**: Mass spectra of the triply charged hACE2 tryptic peptide 466-475 in the glycated (panel A) and non-glycated (panel B) form. **Figure SIV**: Mass spectra of the triply charged hACE2 tryptic peptide 560-577 in the glycated (panel A) and non-glycated (panel B) form. **Figure SV**: Mass spectra of the triply charged hACE2 tryptic peptide 601-621 in the glycated (panel A) and non-glycated (panel B) form. **Figure SVI**: Mass spectra of the triply charged hACE2 tryptic peptide 626-644 in the glycated (panel A) and non-glycated (panel B) form. **Figure SVII**: Mass spectra of the triply charged hACE2 tryptic peptide 658-671 in the glycated (panel A) and non-glycated (panel B) form. **Figure SVIII**: Mass spectra of the triply charged hACE2 tryptic peptide 679-697 in the glycated (panel A) and non-glycated (panel B) form. **Figure SIX**: hACE2 PNGase F deglycosylation. SDS-PAGE performed on 500 nanograms of hACE2 digested with five units of PNGase F (Promega, catalog n. V4831) overnight at 37 °C and then analyzed on a NuPAGE 4-12 Bis-Tris gel (Thermo, catalog no. NP326) using a NuPAGE MES running buffer (Thermo, catalog no. NP0002). Lane 1=Bio-Rad Precision Standard (catalog no. 161-0373); lane 2=deglycosylation blank; lane 3=PNGase-digested hACE2; lane 4= undigested hACE2. The band at about 35 KDa in lanes 2 and 3 is the PNGase F. **Figure SX**. Glycated hACE binding to SARS-CoV-2 Spike protein. A, hACE2 control, B, hACE2 at 12 days incubation time with 60 mM and C, 120 mM of glucose. D, Values of kinetic binding parameters of hACE2-SARS-CoV-2 Spike protein obtained from SPR measurement. Binding sensorgrams for SARS-CoV-2 Spike protein and several analyte concentrations of 1 µg of immobilized Spike protein and ACE2. Samples of ACE2 were diluted in HBS-EP+ at different concentration (64 µg/ml, 32µg/ml, 16 µg/ml, 8 µg/ml, and 4 µg/ml) and injected for 120s at a flow rate of 30µl/min on flow cell 3 and 4. Formulation buffer was run as a control. Dissociation was followed for 300sec; regeneration was achieved with a 60-sec pulse of 3M MgCl2. ACE2 concentrations were listed on the right side and were expressed in µg/ml. Ligand-analyte affinity (KD) and kinetic parameters (Association rate, Ka; Dissociation rate, Kd) of Originator and Biosimilar Rituximab was calculated with the Biacore T200 Evaluation Software (version 2.0; GE Healthcare). **Figure SXI**: RBD amino acid sequence; 319–541 residues and crystal structure of SARS-CoV-2 Spike receptor-binding domain (RBD) bound with ACE2 (PDB ID: 6M0J). Lysine 353 (K353) undergoes mild glycation by high-glucose (12 mM) exposure. Asparagine (N).

**Additional file 2: Figure S2**. Mass spectra of the triply charged hACE2 tryptic peptide 342-357 in the glycated (panel A) and non-glycated (panel B) form. hACE2 is for human Angiotensin Converting enzyme 2 type.

**Additional file 3: Figure S3**. Mass spectra of the triply charged hACE2 tryptic peptide 466-475 in the glycated (panel A) and non-glycated (panel B) form. hACE2 is for human Angiotensin Converting enzyme 2 type.

**Additional file 4: Figure S4**. Mass spectra of the triply charged hACE2 tryptic peptide 560-577 in the glycated (panel A) and non-glycated (panel B) form. hACE2 is for human Angiotensin Converting enzyme 2 type.

## Data Availability

Data and materials are available if required.

## Abbreviations

ADA: American Diabetes Association
COVID-19: Coronavirus disease-19
CV: Cardiovascular
DM: Diabetes mellitus
FFPE: Formalin-fixed paraffin-embedded
hACE2: Recombinant human ACE2
HTX: Heart transplantation
ISH: In situ hybridization
Non-DM: Without diabetes mellitus
PBS: Phosphate buffered saline
RBD: Spike receptor‐binding domain
RT: Room temperature
SARS-CoV-2: Severe acute respiratory syndrome
SPR: Surface plasmon resonance
TBS: Tris-buffered saline
TMPRSS2: Transmembrane protease serine protease-2
