# Supplementary material for: Glycated ACE2 receptor in diabetes: open door for SARS-COV-2 entry in cardiomyocyte
Source: Cardiovasc Diabetol. 2021 May 7;20:99. doi: 10.1186/s12933-021-01286-7 (PMC8104461; doi:10.1186/s12933-021-01286-7)
Supplement: Supplementary file 1 — Additional file 1: Figure SI. Immunofluorescence negative control. Sections from diabetic and non-diabetic patients were performed with blocking solution, supplemented with a non-immune immunoglobulin IgG antibody, following by secondary antibody Alexa Fluor 488 or 633 incubation for 1 hour at RT. All samples were stained with DAPI (5 μg/ml) for 10 min before mounting in Vectashield Mounting Medium (Vector Laboratories, Burlingame, CA, USA). All slides were imaged using a Zeiss LSM 710 confocal microscope (Zeiss, Oberkochen, Germany) with a plan apochromat X63 (NA1.4) oil immersion objective. DM: diabetes mellitus; Non-DM: without diabetes mellitus. Table I: Mass accuracy and retention time (RT) of hACE2 tryptic peptides targeted by glycation. The measurement accuracies were reported in parts per million (ppm) and the chromatographic retention times in minutes (mins). Figure SII: Mass spectra of the triply charged hACE2 tryptic peptide 342-357 in the glycated (panel A) and non-glycated (panel B) form. Figure SIII: Mass spectra of the triply charged hACE2 tryptic peptide 466-475 in the glycated (panel A) and non-glycated (panel B) form. Figure SIV: Mass spectra of the triply charged hACE2 tryptic peptide 560-577 in the glycated (panel A) and non-glycated (panel B) form. Figure SV: Mass spectra of the triply charged hACE2 tryptic peptide 601-621 in the glycated (panel A) and non-glycated (panel B) form. Figure SVI: Mass spectra of the triply charged hACE2 tryptic peptide 626-644 in the glycated (panel A) and non-glycated (panel B) form. Figure SVII: Mass spectra of the triply charged hACE2 tryptic peptide 658-671 in the glycated (panel A) and non-glycated (panel B) form. Figure SVIII: Mass spectra of the triply charged hACE2 tryptic peptide 679-697 in the glycated (panel A) and non-glycated (panel B) form. Figure SIX: hACE2 PNGase F deglycosylation. SDS-PAGE performed on 500 nanograms of hACE2 digested with five units of PNGase F (Promega, catalog n. V4831) overnight [file 12933_2021_1286_MOESM1_ESM.docx]

**Glycated ACE2 receptor** **in diabetes: open door for SARS-COV-2 entry in cardiomyocyte**

^1^Nunzia D’Onofrio*, PhD; ^2^Lucia Scisciola*, PhD; ^2^Celestino Sardu, MD, PhD^Ŧ^; ^3^Maria Consiglia Trotta, PhD; ^4^Marisa De Feo, MD; ^5^Ciro Maiello, MD; ^6^Pasquale Mascolo, MD; ^6^Francesco De Micco, MD; ^2^Fabrizio Turriziani, MD; ^7^Emilia Municinò, MD; ^7^Pasquale Monetti, MD; ^7^Antonio Lombardi, MD; ^7^Maria Gaetana Napolitano, MD; ^8^Federica Zito Marino, MD; ^8^Andrea Ronchi, MD; ^2^Vincenzo Grimaldi, MD; ^9^Anca Hermenean, MD; ^2^Maria Rosaria Rizzo, MD, PhD; ^2^Michelangela Barbieri, MD, PhD; ^8^Renato Franco, MD; ^6^Carlo Pietro Campobasso, MD; ^2^Claudio Napoli, MD, PhD; ^7^Maurizio Municinò, MD; ^2, 10^Giuseppe Paolisso, MD; ^1^Maria Luisa Balestrieri, PhD; ^2, 10^Raffaele Marfella, MD PhD.

^1^Department of Precision Medicine, University of Campania L. Vanvitelli, Italy; ^2^Department of Advanced Medical and Surgical Sciences, University of Campania L. Vanvitelli, Italy; ^3^Department of Experimental Medicine, University of Campania "Luigi Vanvitelli", Naples, Italy; ^4^Department of Cardio-Thoracic Sciences, University of Campania “Luigi Vanvitelli”, Naples, Italy; ^5^Unit of Cardiac Surgery and Transplants, AORN Ospedali dei Colli-Monaldi Hospital, 80131 Naples, Italy; ^6^Department of Experimental Medicine Forensic Pathology Service University of Campania L. Vanvitelli, Italy; ^7^Department of Forensic, Evaluative and Necroscopic Medicine ASL Napoli 2 NORD, Italy; ^8^Department of Mental and Physical Health and Preventive Medicine University of Campania L. Vanvitelli, Italy; ^9^Institute of Life Science, Vasile Goldis Western University, Arad, Romania; 10. Mediterranea Cardiocentro, Naples, Italy.

*Authors equally contributed as first author

**Short title:** Diabetes-induced glycation of ACE2 and SARS-COV-2;

**Word count**: 1286;

**Subject code**: ACE2, Receptors; Diabetes, Type 2; Cardiomyopathy;.

Ŧ**Corresponding author**:

Prof. Celestino Sardu,

Piazza Miraglia, 2. 80138 Napoli, Italy

Tel. +39 081 5665110

Fax+39 081 5665303

Email: [celestino.sardu@unicampania.it](mailto:celestino.sardu@unicampania.it)

**DATA SUPPLEMENT**

**Figure I.**


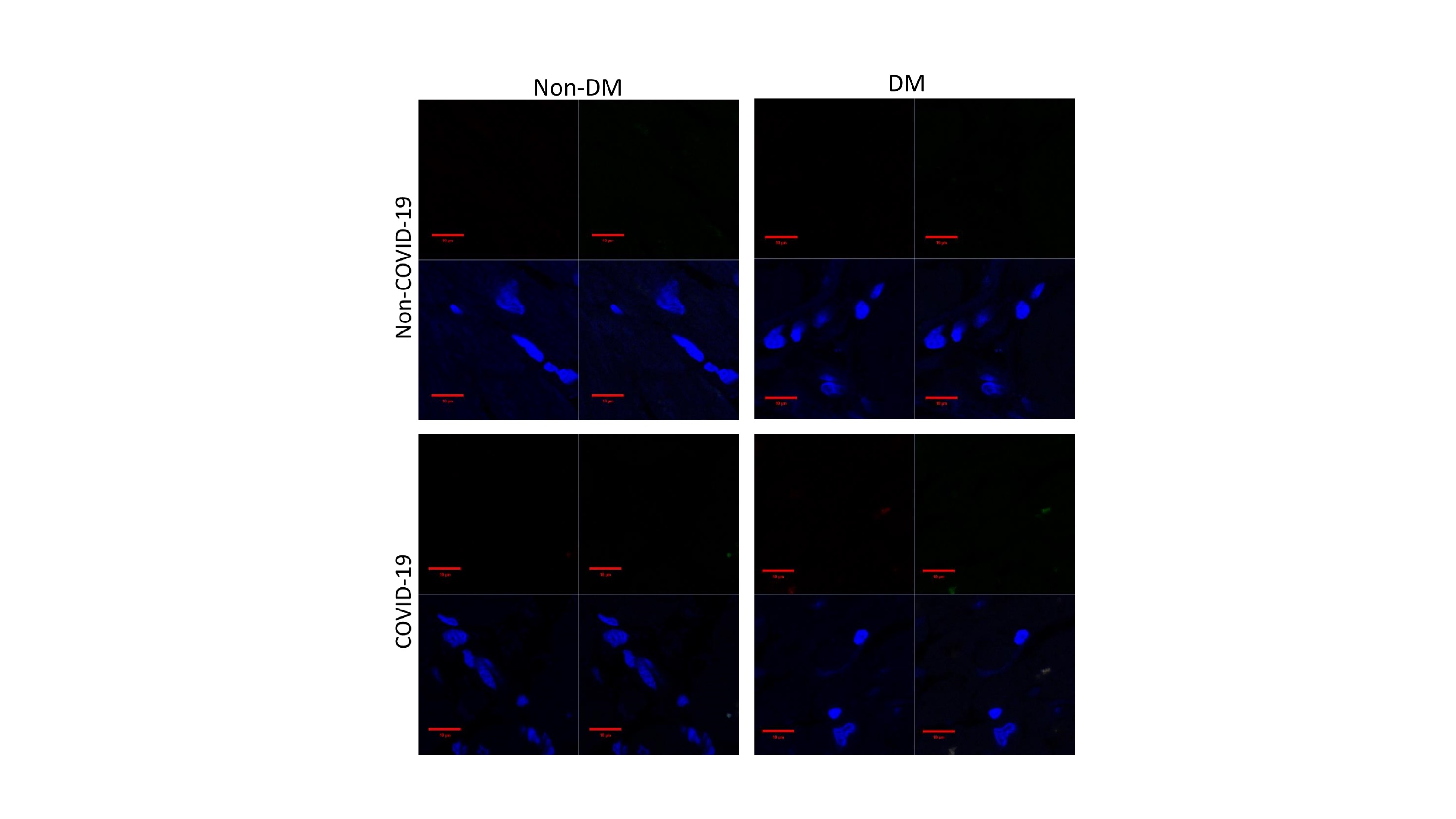


**Online Figure I: Immunofluorescence negative control**. Sections from diabetic and non-diabetic patients were performed with blocking solution, supplemented with a non-immune immunoglobulin IgG antibody, following by secondary antibody Alexa Fluor 488 or 633 incubation for 1 hour at RT. All samples were stained with DAPI (5 μg/ml) for 10 min before mounting in Vectashield Mounting Medium (Vector Laboratories, Burlingame, CA, USA). All slides were imaged using a Zeiss LSM 710 confocal microscope (Zeiss, Oberkochen, Germany) with a plan apochromat X63 (NA1.4) oil immersion objective. DM: diabetes mellitus; Non-DM: without diabetes mellitus.

| **Sequence** | **Accuracy**  **(ppm)** | **Accuracy glycated**  **(ppm)** | **RT**  **(min)** | **RT glycated (min)** | **Sequence Span** |
| --- | --- | --- | --- | --- | --- |
|  |  |  |  |  |  |
| **Glucose-12 mM** |  |  |  |  |  |
| AVCHPTAWDLGKGDFR | 8.44 | 0.00 | 16.62 | 18.54 | 342-357 |
| NSFVGWSTDWSPYADQSIKVR | 0.38 | 0.08 | 26.53 | 26,58 | 601-621 |
| SALGDKAYEWNDNEMYLFR | 0.04 | 0.04 | 26.39 | 26.18 | 626-644 |
| VKNQMILFGEEDVR | 1.03 | 1.13 | 20,77 | 20,65 | 658-671 |
| ISFNFFVTAPKNVSDIIPR | 0.56 | 0.60 | 30.80 | 30.60 | 679-697 |
|  |  |  |  |  |  |
| **Glucose-60 mM** |  |  |  |  |  |
| AVCHPTAWDLGKGDFR | -10.55 | 0.82 | 21.28 | 21.3 | 342-357 |
| GEIPKDQWMK | -1,44 | -0.24 | 16.78 | 16.9 | 466-475 |
| LGKSEPWTLALENVVGAK | -1.25 | 0.31 | 30.70 | 30.47 | 560-577 |
| NSFVGWSTDWSPYADQSIKVR | -0.61 | 1.23 | 29,44 | 29.27 | 601-621 |
| SALGDKAYEWNDNEMYLFR | 0,00 | 0,13 | 29,43 | 29,14 | 626-644 |
| VKNQMILFGEEDVR | -3.32 | 0.36 | 23.79 | 23.56 | 658-671 |
| ISFNFFVTAPKNVSDIIPR | -1.98 | 1.39 | 33.64 | 33.4 | 679-697 |
|  |  |  |  |  |  |
| **Glucose-120 mM** |  |  |  |  |  |
| AVCHPTAWDLGKGDFR | -3.26 | -3.55 | 21.27 | 21.12 | 342-357 |
| GEIPKDQWMK | -2.73 | -1.14 | 16.78 | 16.67 | 466-475 |
| LGKSEPWTLALENVVGAK | 0.14 | -1.10 | 30.7 | 30.47 | 560-577 |
| NSFVGWSTDWSPYADQSIKVR | -0.61 | -0.41 | 29.47 | 29.37 | 601-621 |
| SALGDKAYEWNDNEMYLFR | -0.93 | 0.17 | 29.43 | 29.27 | 626-644 |
| VKNQMILFGEEDVR | -1.36 | -3.28 | 23.73 | 23.56 | 658-671 |
| ISFNFFVTAPKNVSDIIPR | -1.25 | -1.89 | 33.63 | 33.4 | 679-697 |

**Online Table I**: Mass accuracy and retention time (RT) of hACE2 tryptic peptides targeted by glycation. The measurement accuracies were reported in parts per million (ppm) and the chromatographic retention times in minutes (mins).


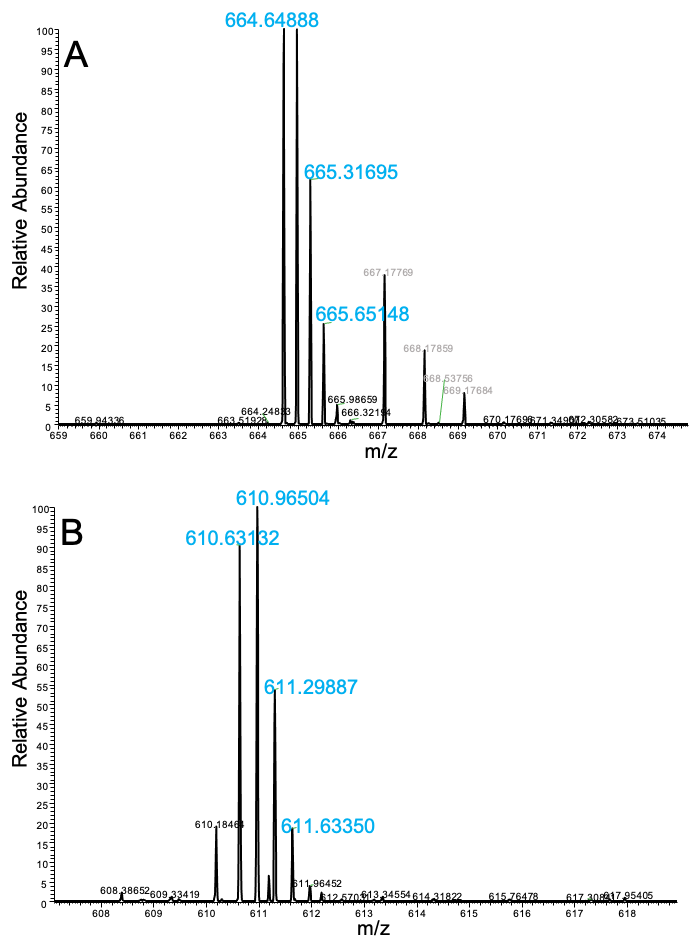


**Online Figure II**: Mass spectra of the triply charged hACE2 tryptic peptide 342-357 in the glycated (panel A) and non-glycated (panel B) form.


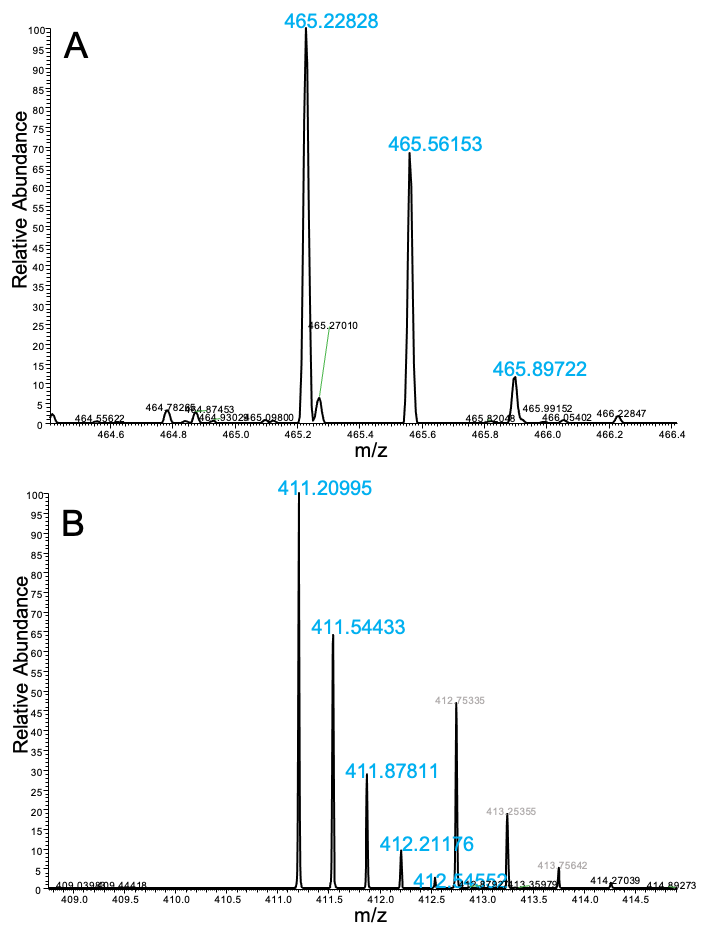


**Online Figure III**: Mass spectra of the triply charged hACE2 tryptic peptide 466-475 in the glycated (panel A) and non-glycated (panel B) form.


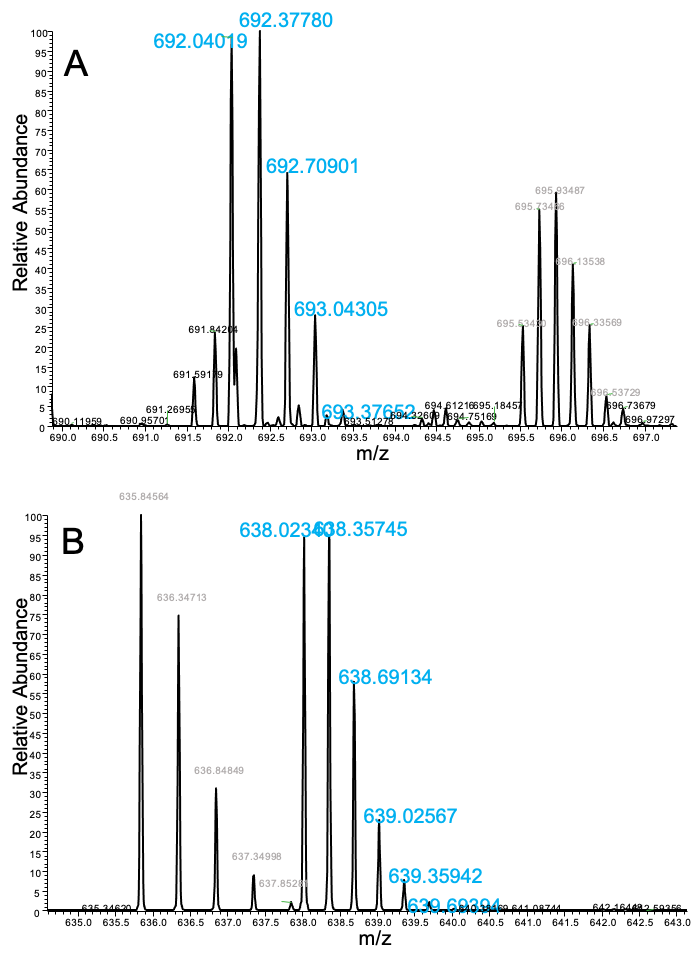


**Online Figure IV**: Mass spectra of the triply charged hACE2 tryptic peptide 560-577 in the glycated (panel A) and non-glycated (panel B) form.


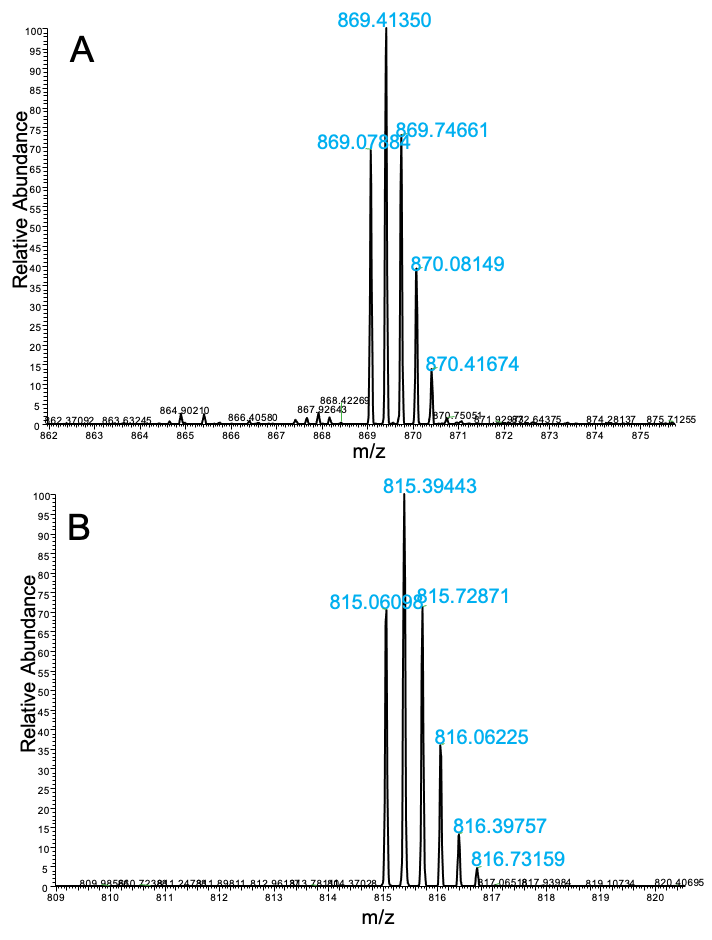


**Online Figure V**: Mass spectra of the triply charged hACE2 tryptic peptide 601-621 in the glycated (panel A) and non-glycated (panel B) form.


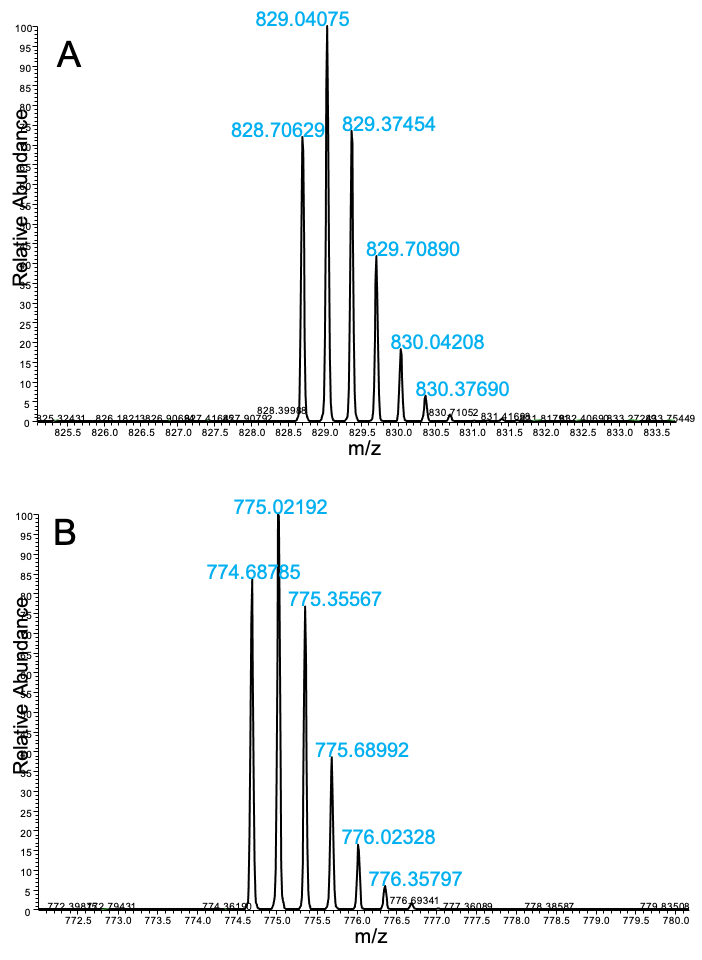


**Online Figure VI**: Mass spectra of the triply charged hACE2 tryptic peptide 626-644 in the glycated (panel A) and non-glycated (panel B) form.


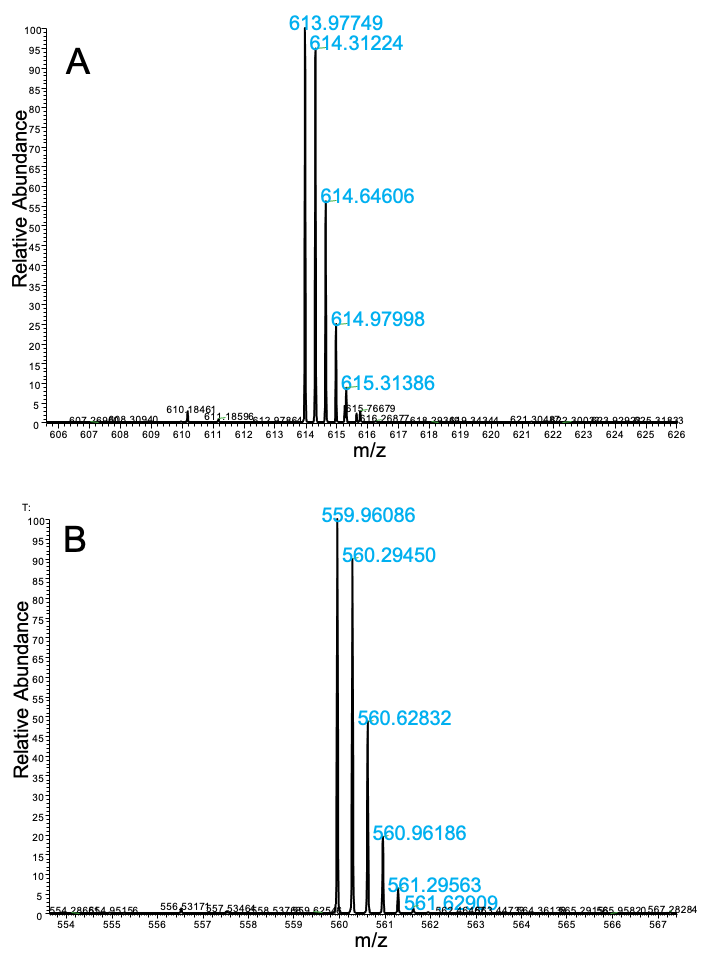


**Online Figure VII**: Mass spectra of the triply charged hACE2 tryptic peptide 658-671 in the glycated (panel A) and non-glycated (panel B) form.


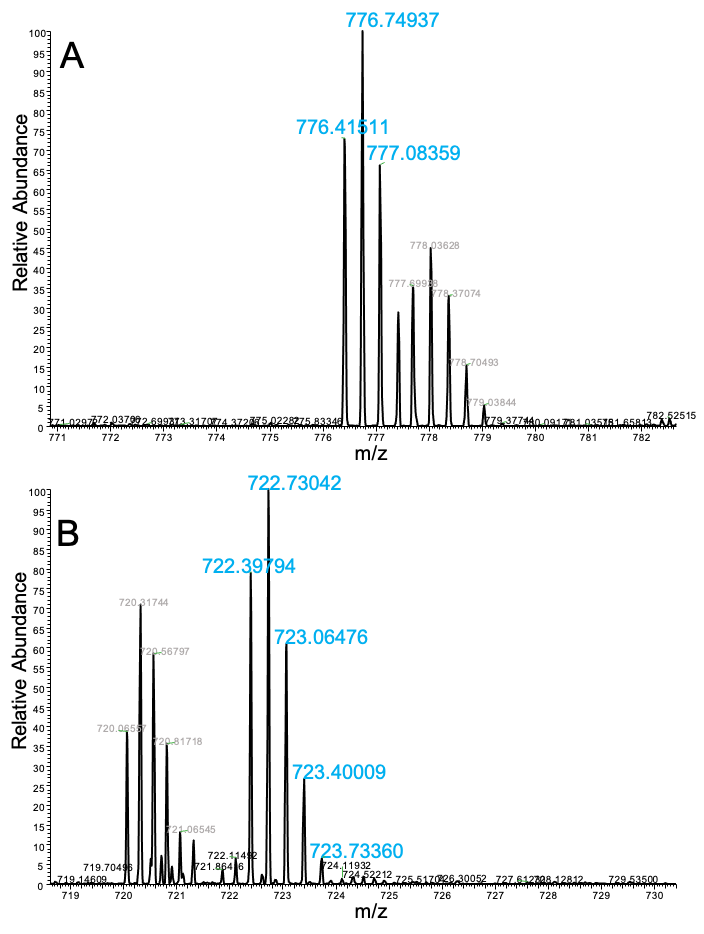


**Online Figure VIII**: Mass spectra of the triply charged hACE2 tryptic peptide 679-697 in the glycated (panel A) and non-glycated (panel B) form.


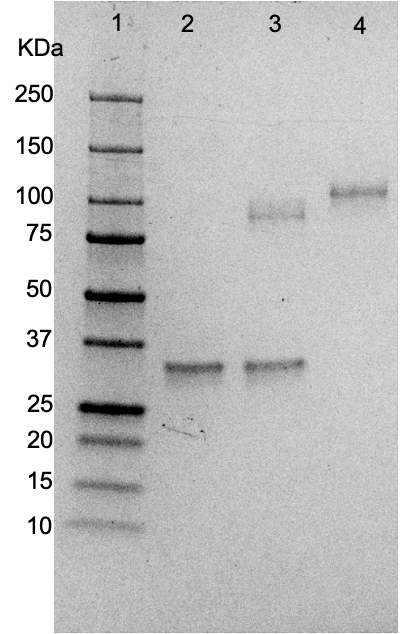


**Online Figure IX**: **hACE2 PNGase F deglycosylation.**

SDS-PAGE performed on 500 nanograms of hACE2 digested with five units of PNGase F (Promega, catalog n. V4831) overnight at 37 °C and then analyzed on a NuPAGE 4-12 Bis-Tris gel (Thermo, catalog no. NP326) using a NuPAGE MES running buffer (Thermo, catalog no. NP0002). Lane 1=Bio-Rad Precision Standard (catalog no. 161-0373); lane 2=deglycosylation blank; lane 3=PNGase-digested hACE2; lane 4= undigested hACE2. The band at about 35 KDa in lanes 2 and 3 is the PNGase F.


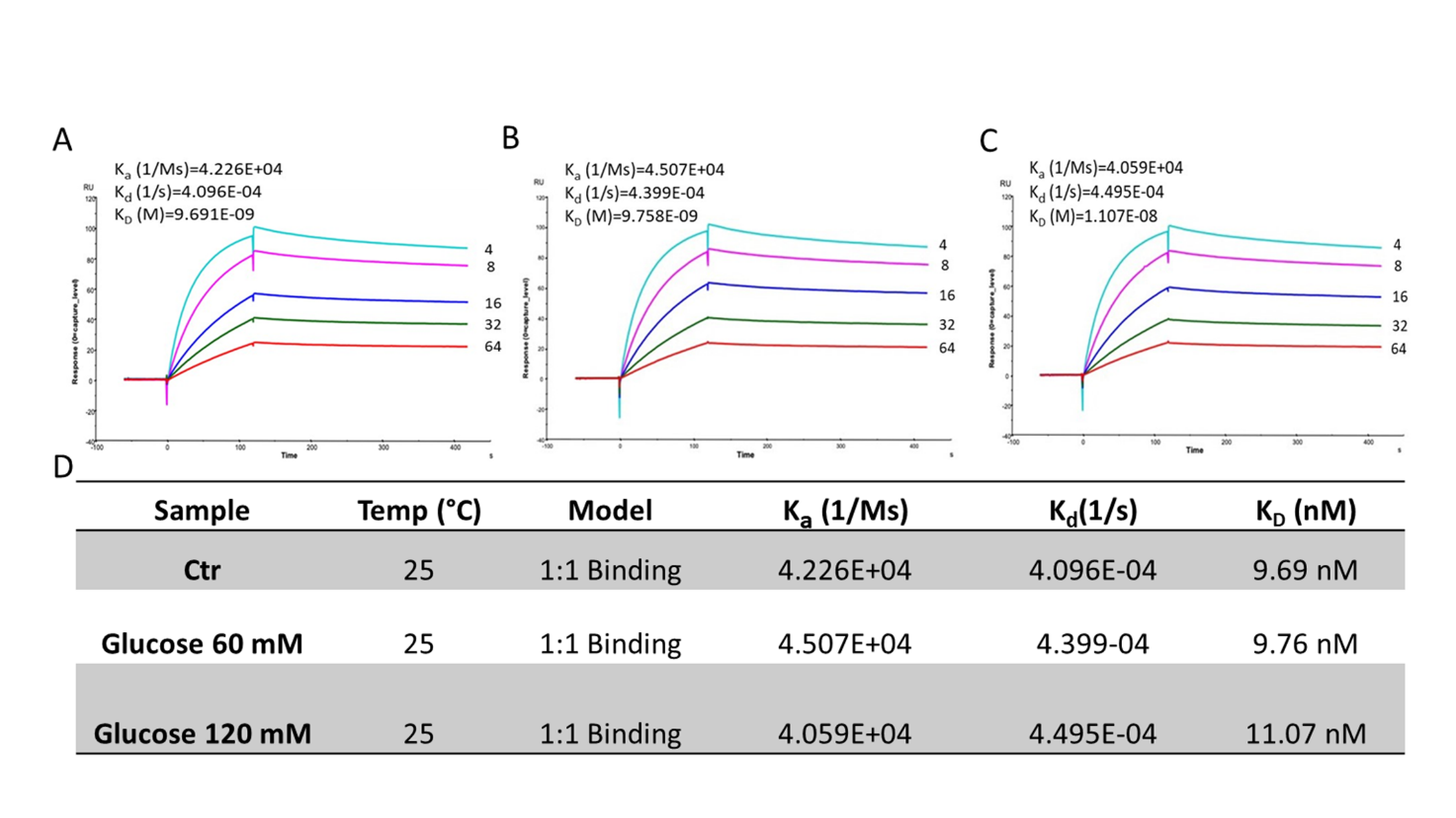


**Online Figure X. Glycated hACE binding to SARS-CoV-2 Spike protein. A**, hACE2 control, **B**, hACE2 at 12 days incubation time with 60 mM and **C**, 120 mM of glucose. **D**, Values of kinetic binding parameters of hACE2-SARS-CoV-2 Spike protein obtained from SPR measurement. Binding sensorgrams for SARS-CoV-2 Spike protein and several analyte concentrations of 1 µg of immobilized Spike protein and ACE2. Samples of ACE2 were diluted in HBS-EP+ at different concentration (64 µg/ml, 32µg/ml, 16 µg/ml, 8 µg/ml, and 4 µg/ml) and injected for 120s at a flow rate of 30µl/min on flow cell 3 and 4. Formulation buffer was run as a control. Dissociation was followed for 300sec; regeneration was achieved with a 60-sec pulse of 3M MgCl2. ACE2 concentrations were listed on the right side and were expressed in µg/ml. Ligand-analyte affinity (KD) and kinetic parameters (Association rate, Ka; Dissociation rate, Kd) of Originator and Biosimilar Rituximab was calculated with the Biacore T200 Evaluation Software (version 2.0; GE Healthcare)


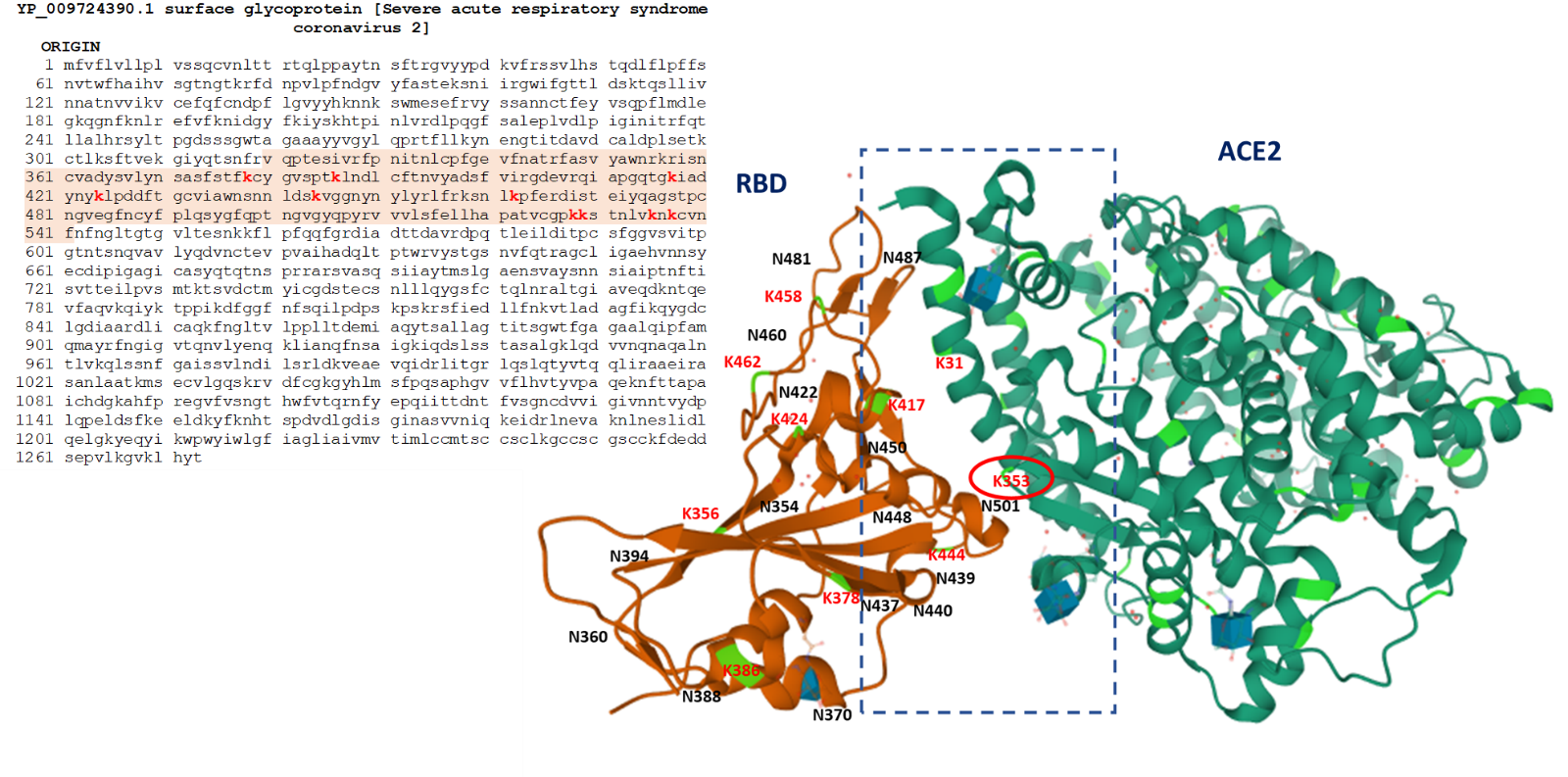


**Online Figure XI**: RBD amino acid sequence; 319–541 residues and crystal structure of SARS-CoV-2 Spike receptor-binding domain (RBD) bound with ACE2 (PDB ID: 6M0J). Lysine 353 (K353) undergoes mild glycation by high-glucose (12 mM) exposure. Asparagine (N).
